# A Practical and Sustainable Ni/Co-Free High-Energy Electrode Material: Nanostructured LiMnO_2_

**DOI:** 10.1021/acscentsci.4c00578

**Published:** 2024-08-26

**Authors:** Yuka Miyaoka, Takahito Sato, Yuna Oguro, Sayaka Kondo, Koki Nakano, Masanobu Nakayama, Yosuke Ugata, Damian Goonetilleke, Neeraj Sharma, Alexey M. Glushenkov, Satoshi Hiroi, Koji Ohara, Koji Takada, Yasuhiro Fujii, Naoaki Yabuuchi

**Affiliations:** † Department of Chemistry and Life Science, Yokohama National University, 79-5 Tokiwadai, Hodogaya-ku, Yokohama, Kanagawa 240-8501, Japan; ‡ Department of Applied Chemistry, Tokyo Denki University, 5 Senju Asahi-Cho, Adachi, Tokyo 120-8551, Japan; § Frontier Research Institute for Materials Science (FRIMS), Nagoya Institute of Technology, Gokiso-cho, Showa-ku, Nagoya, Aichi 466-8555, Japan; ∥ Advanced Chemical Energy Research Center, Institute of Advanced Sciences, Yokohama National University, Yokohama 240-0067, Japan; ⊥ School of Chemistry, University of New South Wales, Sydney, NSW 2052, Australia; # Research School of Chemistry, The Australian National University, Canberra, ACT 2600, Australia; 7 Faculty of Materials for Energy, Shimane University, Matsue, Shimane 690-8504, Japan; 8 Tosoh Corporation, 4560 Kaisei-cho, Shunan-Shi, Yamaguchi 746-8501, Japan

## Abstract

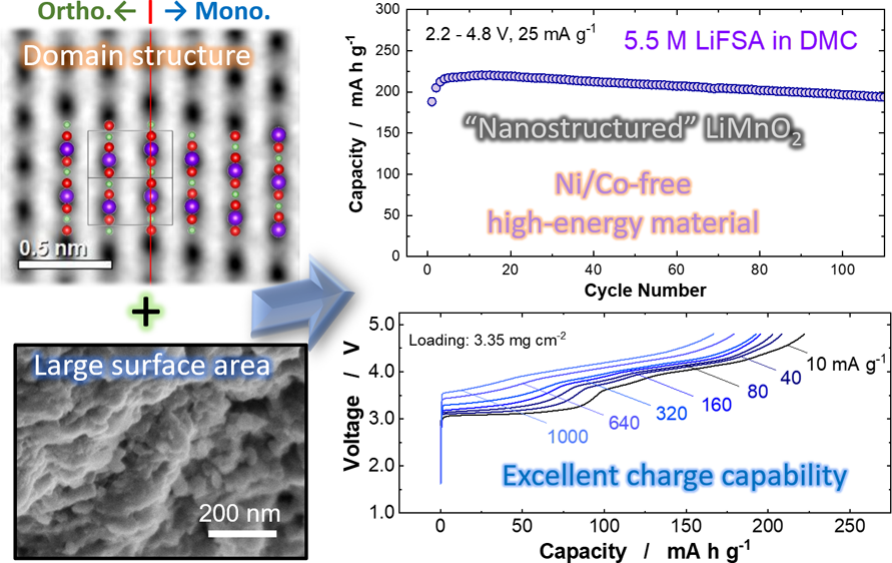

Ni/Co-free high-energy positive electrode materials are of great importance to ensure the sustainability of Li-ion battery production and its supply chain in addition to minimizing environmental impact. Here, nanostructured LiMnO_2_ with both orthorhombic/monoclinic layered domains is synthesized, and its lithium storage properties and mechanism are examined. High-energy mechanical milling is used to convert the metastable and nanosized LiMnO_2_ adopting the cation-disordered rocksalt structure to an optimal domain-segregated layered LiMnO_2_. This positive electrode produces an energy density of 820 W h kg^–1^, achieved by harnessing a large reversible capacity with relatively small voltage hysteresis on electrochemical cycles. Moreover, voltage decay for cycling, as observed for Li-excess Mn-based electrode materials, is effectively mitigated. Furthermore, by determining the structure–property relationships of different LiMnO_2_ polymorphs, LiMnO_2_ with similar domain structure and surface area is successfully synthesized with an alternative and simpler method, without the metastable precursor and high-energy mechanical milling. The cyclability of domain-containing LiMnO_2_ is also improved with the use of a highly concentrated electrolyte coupled with a lithium phosphate coating due to the suppression of Mn dissolution. These findings maximize the possibility of the development of high-energy, low-cost, and practical rechargeable batteries made from sustainable and abundant Mn sources without Ni/Co.

## Abstract 2

Domain-structured LiMnO_2_ with large surface area has been synthesized and proposed as Co/Ni-free positive electrode materials with high-energy density for practical Li-ion battery applications.

## Introduction

The electrification of worldwide mobility solutions is effectively a prerequisite to minimize dependence on fossil fuels as energy resources. Among rechargeable energy storage devices, Li-ion batteries provide the highest gravimetric and volumetric energy density, and the technology has been optimized and heavily researched in the past three decades since its first commercialization in 1991. Now, Li-ion batteries are able to provide power for electric vehicles, which has in part been enabled by the significant decline in their cost and continual improvements in energy density.^1^ Although the global market of electric vehicles is rapidly growing, further reduction of battery cost is necessary to allow for further penetration of electric vehicles into the automobile market. The price of reagents and materials becomes critical as the cost of batteries decreases,^2^ and the positive electrode is the most expensive component in Li-ion batteries.^3^ Spinel-type LiMn_2_O_4_ and olivine-type LiFePO_4_ have been studied and practically used as cost-effective positive electrode materials, but the energy density of these low-cost materials is limited to approximately 500 W h kg^–1^ based on Li/Li^+^ which is significantly lower than that of Ni-based layered materials (∼750 W h kg^–1^). Consequently, Ni-based layered materials with cationic redox reaction of Ni ions, i.e., Ni^2+^/Ni^3+^/Ni^4+^, are mainly used in Li-ion batteries for state-of-the-art electric vehicles, which result in extended cruising distances. Ni-based layered materials, derivatives of LiNiO_2_, e.g., LiNi_1–*x*–*y*_Mn_*x*_Co_*y*_O_2_,^4−8^ are often plagued by the relatively high cost of Co and Ni compounds due to their limited abundance in the crust and the mining/processing steps involved to make battery grade electrodes. Moreover, the cost of Ni is increasing as the electric vehicle market expands, and a high price risk or volatility is anticipated in the future, not to mention supply chain challenges.^9^ Therefore, the development of Ni-/Co-free high-energy positive electrode materials is desired to further reduce the cost of Li-ion batteries and to ensure its sustainability.

Recently, Li_2_MnO_3_-based electrode materials with a layered structure and its derivatives have been extensively studied as potential high-energy and low-cost positive electrode materials. Higher energy density, ∼900 W h kg^–1^, can be realized using Li_2_MnO_3_-based electrode materials with anionic redox reaction, whereby the Li extracted from host structures is compensated for by negatively charged oxygen species. Nevertheless, reversibility of anionic redox is insufficient, and gradual oxygen loss upon cycling results in the lowering of operating voltage.^10,11^ Large voltage hysteresis on charge–discharge for anionic redox is another practical issue. In addition, Mn-based Li-excess oxides with a rocksalt structure have also been reported, and an even higher energy density of 1000–1100 W h kg^–1^ has been demonstrated.^12,13^ However, similar problems to the layered structure are noted for these rocksalts, cyclability, and voltage hysteresis. Such disadvantages of these electrode materials currently hinder their use in practical applications, and therefore, further stabilization of anionic redox and the mitigation of voltage loss are necessary.

Among the Mn-based electrode materials, as a counterpart of LiCoO_2_ and LiNiO_2_, stoichiometric LiMnO_2_ was also extensively studied as a positive electrode material.^14−18^ Thermodynamically stable LiMnO_2_ crystallizes in a so-called “zigzag”-type layered structure with orthorhombic space-group symmetry.^14^ During electrochemical cycling, a particularly unusual phase transition is noted where a spinel-like phase with low crystallinity is eventually formed.^17^ Unlike the more conventional phase transitions noted in intercalation compounds, where on either charge or discharge, Li extraction or insertion in the case of the positive electrode, a phase transition is noted, e.g., from hexagonal LiCoO_2_ to monoclinic Li_∼0.5_CoO_2_, the transition in LiMnO_2_ to the spinel-like phase is significantly more gradual and occurs with cycling coupled with Mn migration, over a number of charge/discharge cycles. This implies a kinetic inhibition to this phase transition.

In LiMnO_2_ reversible capacities increase to ∼220 mA h g^–1^ at 8.3 mA g^–1^, after the formation of the spinel-like phase.^19^ Nevertheless, the phase transition kinetics are quite slow, and over 30 cycles are required to obtain such a large reversible capacity, which is likely associated with the formation of the spinel-like phase and its reversible cycling. Moreover, energy efficiency is low, and approximately 20% of the energy density is lost, presumably associated with the inferior electrode kinetics. Therefore, there is a coupled relation between the generation of the spinel-like phase and optimal electrochemical performance.

Another polymorph of LiMnO_2_ adopting monoclinic space group symmetry with a layered structure but metastable was prepared by a soft chemistry route from NaMnO_2_ and delivered *ca*. 180 mA h g^–1^ even after 100 cycles.^20^ However, initial Coulombic efficiency is limited to only 70% and a long activation process (coupled with the increase in reversible capacity) over 40 cycles is required to attain high reversible capacities. The phase stability and reversibility of LiMnO_2_ appear to be correlated and synthesizing the appropriate phase or composite with high reversibility may be the answer to an excellent electrode material.

In this article, nanostructured LiMnO_2_, which contains both orthorhombic and monoclinic layered domains, is synthesized, and its lithium storage properties are systematically examined. First, metastable and nanosized LiMnO_2_ with a rocksalt structure is prepared by mechanical milling,^21^ and this sample is used as a precursor. Heat-treatment of rocksalt LiMnO_2_, which is energetically unstable compared with orthorhombic and monoclinic layered phases, results in the crystallization of LiMnO_2_ with the unique nanostructure. These nanostructures influence phase transition processes, resulting in a spinel-like phase with electrochemical cycling. The grain size remains comparable to that of metastable rocksalt LiMnO_2_ (∼150 nm) prepared by mechanical milling, which is found to be essential in producing a high-performance electrode material without Ni/Co. Moreover, these findings and understanding for different LiMnO_2_ polymorphs permit the direct synthesis of LiMnO_2_ with a similar nanostructure without the use of the metastable precursor and high-energy ball milling. From these results, the possibility of developing practical Ni/Co-free high-energy positive electrode materials is both discussed and demonstrated.

## Results and Discussion

### Synthesis of Nanostructured LiMnO_2_ and Structural Characterization

**Figure 1:**
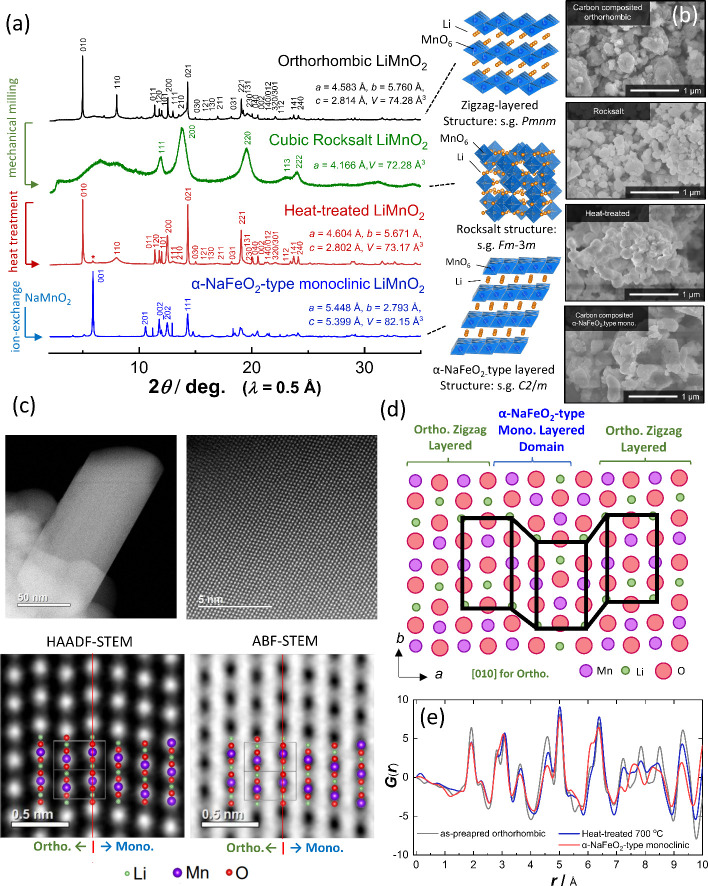
Synthesis of four different LiMnO_2_ polymorphs: (a) XRD patterns and schematic illustrations of crystal structures, (b) SEM images, (c) STEM images of heat-treated LiMnO_2_, (d) schematic illustration of domain structures for heat-treated LiMnO_2_, and (e) pair distribution functions of orthorhombic, heat-treated and monoclinic LiMnO_2_. Schematic illustrations of crystal structures were drawn using the VESTA program.^61^

The precursor metastable and nanosized LiMnO_2_ with a cubic rocksalt structure was prepared by mechanical milling of a zigzag layered LiMnO_2_ with orthorhombic symmetry, as described in the Experimental Methods, using a process which has been previously detailed in the literature.^21^ Synchrotron X-ray diffraction (XRD) patterns of a thermodynamically stable phase, orthorhombic LiMnO_2_, and metastable phase, cubic rocksalt LiMnO_2_, are compared in Figure 1a. The reflections for the orthorhombic phase are completely lost after mechanical milling, and broad reflections, which can be assigned to the cubic phase, are observed. Crystalline sizes of cubic LiMnO_2_ range from 3 to 5 nm based on Scherrer analysis.^21^ In addition, the particle morphology from orthorhombic to rocksalt LiMnO_2_ is completely changed as observed by field emission scanning electron microscopy (FE-SEM) in Figure 1b and Supporting Figure S1a. Large particle sizes, ∼10 μm, for orthorhombic LiMnO_2_ are effectively reduced to submicron scale, which consists of smaller grains, 50–150 nm (Figure 1b).

Heat-treatment of metastable cubic rocksalt LiMnO_2_ at 700 °C results in the formation of LiMnO_2_ with a different crystal structure. Note, all reflections after heat-treatment can be assigned to the orthorhombic phase, as shown in Figure 1a, but intensity and area (peak shapes) of reflections are clearly different for as prepared orthorhombic LiMnO_2_ and ball-milled and heat-treated LiMnO_2_. For instance, the reflection intensity of (110) at 2θ ∼ 8° is lower whereas peak intensity of (021) at 2θ ∼ 14.3° is higher in the ball-milled and heat-treated LiMnO_2_ relative to as-prepared LiMnO_2_ (Supporting Figure S1b). This observation is likely to originate from structural (dis)ordering in the orthorhombic phase. Similar intensity fluctuations are noted for LiMnO_2_ synthesized by the hydrothermal method^22^ and the samples synthesized with lithium deficiency.^23^

For comparison, another metastable polymorph of LiMnO_2_ was prepared by ion-exchange from NaMnO_2_ with the in-plane distorted α-NaFeO_2_-type layered structure,^15^ and its synchrotron XRD diffraction pattern is also shown in Figure 1a. Mn^3+^ is a typical Jahn–Teller ion, so in-plane distortion for transition metal layers is induced, leading to a monoclinic lattice. At 2θ ∼ 6° the most intense reflection, (001) is clearly observed for monoclinic layered LiMnO_2_ and it is important to note that the small reflection at the same 2θ position is also observed in ball-milled and heat-treated LiMnO_2_. Both monoclinic and orthorhombic phases consist of common oxygen packing, a cubic-close-packed structure (Supporting Figure S1c), and only arrangements of cations are different at the octahedral sites. To examine the origin of the structural (dis)order, the formation energy of LiMnO_2_ with three different polymorphs, orthorhombic zigzag layered, monoclinic layered, and cubic rocksalt structures, is computed by density functional theory (DFT) calculations, and the results are summarized in Supporting Figure S2. The energy of cubic rocksalt LiMnO_2_ is unstable by 50 and 64 meV per atom when compared with the monoclinic and orthorhombic phases, respectively. XRD data show evidence of the nucleation of the orthorhombic phase on heating of rocksalt LiMnO_2_. However, a growth rate of the orthorhombic phase along the *b*/*c*-axis directions would be faster than or different to that of *a*-axis direction, leading to the formation of planar defects. This would be associated with the mismatch of the Li and Mn sites and thus would not form the ideal zigzag layered structure on growth in different directions or domains. This mismatch locally results in the formation of domains of the α-NaFeO_2_-type monoclinic layered structure as shown in Figure 1d. In addition, the energy difference between monoclinic layered and orthorhombic phases is relatively small; therefore, the possibility of nucleation and growth of the monoclinic phase cannot be eliminated, especially if it is seeded at the planar defect sites described above. Indeed, the small (001) at 2θ ∼ 6°, which is not observed for the pure orthorhombic phase, is detected for the ball-milled and heat-treated LiMnO_2_. The presence of planar defects is directly visualized by high-angle annular dark-field scanning transmission electron microscopy (HAADF-STEM) as shown in Figure 1c and Supporting Figure S3. Different structural domains are clearly visualized in the STEM images, and the domain size for the orthorhombic phase is relatively large, as shown in Supporting Figure S3. The presence of Li and O is also partly observed in annular bright-field (ABF) STEM images. Similar structural (dis)ordering is also observed for NaMnO_2_, and such planar defects are also regarded as the presence of multiple twinning in particles.^24^

To further validate the planar defects in the heat-treated sample, XRD patterns with different layered domains were simulated using the DIFFaX program,^25^ and similar analysis was also conducted for the previously reported Li-deficient phase.^15^ The fraction of monoclinic layered domains was continuously altered from 0 to 100% versus orthorhombic layered domains, and XRD patterns were simulated for these conditions (Supporting Figure S4). The observed XRD pattern, including (110) with the broad profile, is successfully reproduced when 30% of the monoclinic layered domains coexists with the orthorhombic structure. This finding is further supported by total X-ray total scattering study where high-energy X-ray scatting data at *E* = 61.4 keV were collected and the corresponding experimental X-ray pair distribution function is shown in Supporting Figure S5. The pair distribution function of the ball-milled and heat-treated LiMnO_2_ is reminiscent of both orthorhombic and monoclinic phases and can be considered an average profile or between these two structures, as compared in Figure 1e. All structural analysis conducted in this study concludes that ball-milled and heat-treated LiMnO_2_ has both the orthorhombic zigzag layered and α-NaFeO_2_-type monoclinic layered domains. Note that grain sizes of the sample after heat treatment at 700 °C remain comparable to cubic rocksalt LiMnO_2_, approximately 100–150 nm (Figure 1b), and smoothly faceted grains are found after heat-treatment without significant reduction of surface area.

### Electrode Properties of Nanostructured LiMnO_2_

**Figure 2:**
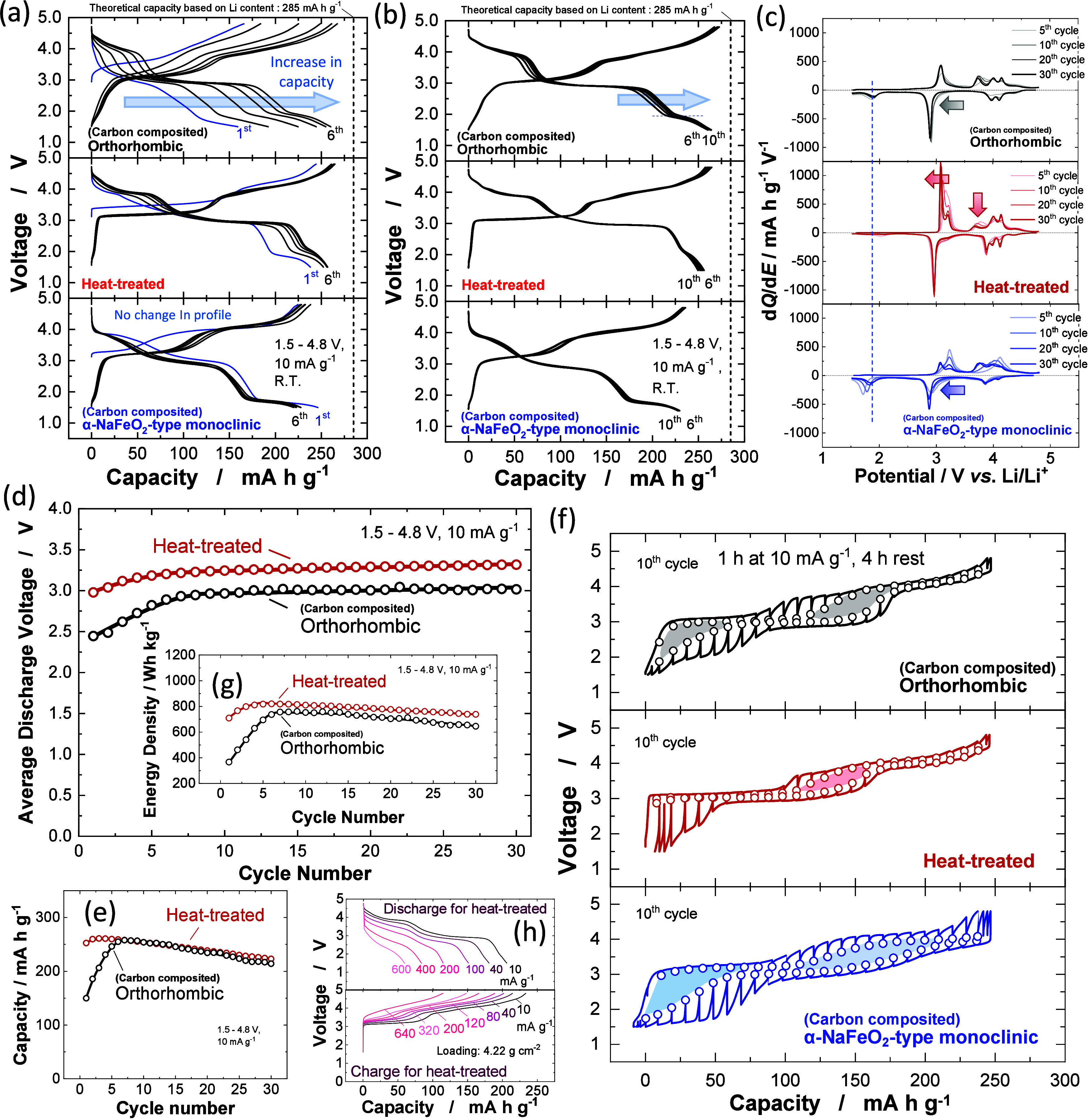
Electrochemistry of different LiMnO_2_ polymorphs: Galvanostatic charge/discharge curves where (a) shows 1st–5th cycles and (b) 6th–10th cycles, (c) differential capacity plots, (d) changes in average discharge voltage, (e) capacity retention, (f) quasi-open circuit voltage for the 10th cycle, (g) energy density variations, and (h) discharge/charge rate capability of heat-treated LiMnO_2_.

Lithium storage properties of LiMnO_2_ obtained by ball-milling and heat treatment of metastable cubic rocksalt LiMnO_2_ were examined. Results are also compared with different LiMnO_2_ polymorphs and pure orthorhombic and monoclinic layered structures. The particle size of as-prepared orthorhombic LiMnO_2_ is large and this adversely impacts performance, therefore this sample was mixed with acetylene black (10 wt % in a mass ratio) and ball milled to reduce the particle size (Supporting Figure S1). The particle size of the sample is effectively reduced by ball milling, and electrode performance is also significantly improved as shown in Supporting Figure S6a. Note this sample remains orthorhombic, as the ball milling conditions were optimized to minimize or avoid the formation of the cubic rocksalt LiMnO_2_. Similarly, a carbon composited electrode was also prepared from monoclinic layered LiMnO_2_, and the electrode performance of the as-prepared sample is shown in Supporting Figure S6b. Charge/discharge curves of these three different LiMnO_2_ polymorphs are shown for the initial 6 cycles and for 6–10th cycles in Figure 2a and 2b, respectively. The data of orthorhombic and monoclinic LiMnO_2_ were obtained from the carbon-composite samples while the as-prepared sample was used for ball-milled and heat-treated LiMnO_2_, without ball milling with acetylene black. The initial charge capacity reaches 245 mA h g^–1^ for the ball-milled and heat-treated sample, which is clearly larger than the as-prepared orthorhombic sample.^19,26^ Moreover, the initial discharge capacity reaches 235 mA h g^–1^ for the ball-milled and heat-treated sample. Reversible capacities increase in the initial several cycles for both samples, characteristic for LiMnO_2_ and are typically associated with a phase transition into a spinel-like phase. The largest reversible capacity is observed at third and eighth cycles for the heat-treated and carbon composited orthorhombic samples respectively on electrochemical cycling at a rate of 10 mA g^–1^. In contrast, the largest reversible capacity of 245 mA h g^–1^ for monoclinic layered LiMnO_2_ is observed at the initial cycle, and no further increase in reversible capacity is evidenced for continuous cycles. No increase in reversible capacity is observed on 6–10th cycles for the heat-treated and monoclinic samples (Figure 1b), suggesting that phase transition kinetics to the spinel-like phase on electrochemical cycling are faster when compared with those of orthorhombic LiMnO_2_. Charge/discharge curves for these samples during 30 cycles at 10 mA g^–1^ is shown in Supporting Figure S7, and the d*Q*/d*E* differential capacity plots are compared in Figure 2c. All samples show large reversible capacities, 240–260 mA h g^–1^, with similar capacity retention with continuous cycling until cycle 30. Nevertheless, voltage profiles and thus energy density are completely different for the three samples. The presence of a voltage plateau at 2 V especially for the discharge process is noted for the as-prepared orthorhombic and monoclinic samples. This trend is further visualized in differential capacity plots in Figure 2c. Smaller polarization with a highly reversible 3 V redox reaction is clearly evidenced for the ball-milled and heat-treated sample, and interestingly, polarization is gradually reduced upon electrochemical cycling (Figure 2c). Average voltage changes for the heat-treated and orthorhombic samples are also plotted in Figure 2d. Although discharge capacity is gradually lost on continuous cycling (220 mA h g^–1^ at 30th cycle at a rate of 10 mA g^–1^, Figure 2e for both samples), no decrease in average voltage is noted in Figure 2d. The decrease in average discharge voltage is a critical problem for the Li-rich system, e.g., Li_1.2_Co_0.13_Ni_0.13_Mn_0.54_O_2_ (see Supporting Figure S8), which hinders its use for practical applications. The problem of voltage decay on electrochemical cycling is effectively mitigated for LiMnO_2_ because the large reversible capacity is expected to originate solely from Mn cationic redox without unstable O anionic redox.

### Comparison of Voltage Hysteresis and Energy Density of LiMnO_2_ Polymorphs

As shown in Figure 2c, differential capacity plots reveal that LiMnO_2_ polymorphs show different voltage hysteresis, which influences their energy density as electrode materials. Quasi open-circuit voltages (QOCVs) for different polymorphs are compared in Figure 2f. The cells were charged/discharged for 1 h at a rate of 10 mA g^–1^, and then were rested at open-circuit for 4 h. The largest voltage hysteresis is observed for monoclinic layered LiMnO_2_. Note that the largest voltage hysteresis for monoclinic LiMnO_2_ is observed for the 2–3 V region, which is almost nonexistent for heat-treated LiMnO_2_ after 4 h relaxation. Additional hysteresis is observed in the 3–4 V region for all samples, but the smallest hysteresis is evidenced for heat-treated LiMnO_2_. QOCVs in Figure 2f were collected at the 10th cycle. QOCVs of heat-treated LiMnO_2_ at the fifth cycle is also shown in Supporting Figure S9, and smaller hysteresis is found at 10th cycle. Although three different LiMnO_2_ polymorphs show distinct charge/discharge profiles in Figure 2b, the variations are explained as the difference in electrode kinetics, especially for the 2–3 V region as clearly found in Figure 2f. Such voltage hysteresis observed under nearly equilibrium conditions is also known for spinel Li_1+*x*_Mn_2_O_4_ and changes as a function of particle size. For spinel Li_1+*x*_Mn_2_O_4_ this was suggested to originate from the difference in kinetics of lithiation/delithiation coupled with the distortion of crystal lattice.^27^ Similar phenomena are expected for LiMnO_2_ polymorphs but associated with the spinel-like phase formation with electrochemical cycling and potentially their lithiation/delithiation kinetics. These differences in voltage hysteresis and electrode kinetics result in the highest energy density for heat-treated LiMnO_2_ (Figure 2g) compared to the other polymorphs. The largest energy density is estimated to be 820 W h kg^–1^ for heat-treated LiMnO_2_, which is larger than those of the Ni-rich layered materials (Supporting Figure S10).^28^ Moreover, voltage decay on the electrochemical cycle was not observed for LiMnO_2_. Rate capability of heat-treated LiMnO_2_ is also shown in Figure 2h. Nearly 50% of capacity is lost at a discharge rate of 200 mA g^–1^, corresponding to approximately 0.7 C. The electrode kinetics on discharge are not superior to Ni-rich electrode materials as expected from inferior kinetics on discharge (Figure 2f). Nevertheless, from the voltage relaxation processes, superior kinetics on “charge” are anticipated because of small voltage hysteresis compared with discharge. Indeed, much better rate-capability on charge is obtained, as shown in Figure 2h. Quick charge ability is an important characteristic for upcoming battery applications, especially for electric vehicles, and it is concluded that LiMnO_2_ is a suitable electrode material for this purpose. It is noted that such inferior electrode kinetics on discharge associated with the lattice distortion is expected to lead to the capacity loss at the 3 V region on continuous cycles (Supporting Figure S8e). In contrast, the superior reversibility at the 4 V region results in better capacity retention, leading to gradual increase for average discharge voltage (Figure 2d and Supporting Figure S8h). Voltage decay observed for Li-rich Mn-based oxides is, therefore, not observed for LiMnO_2_.

### Reaction Mechanisms of Nanostructured LiMnO_2_

**Figure 3:**
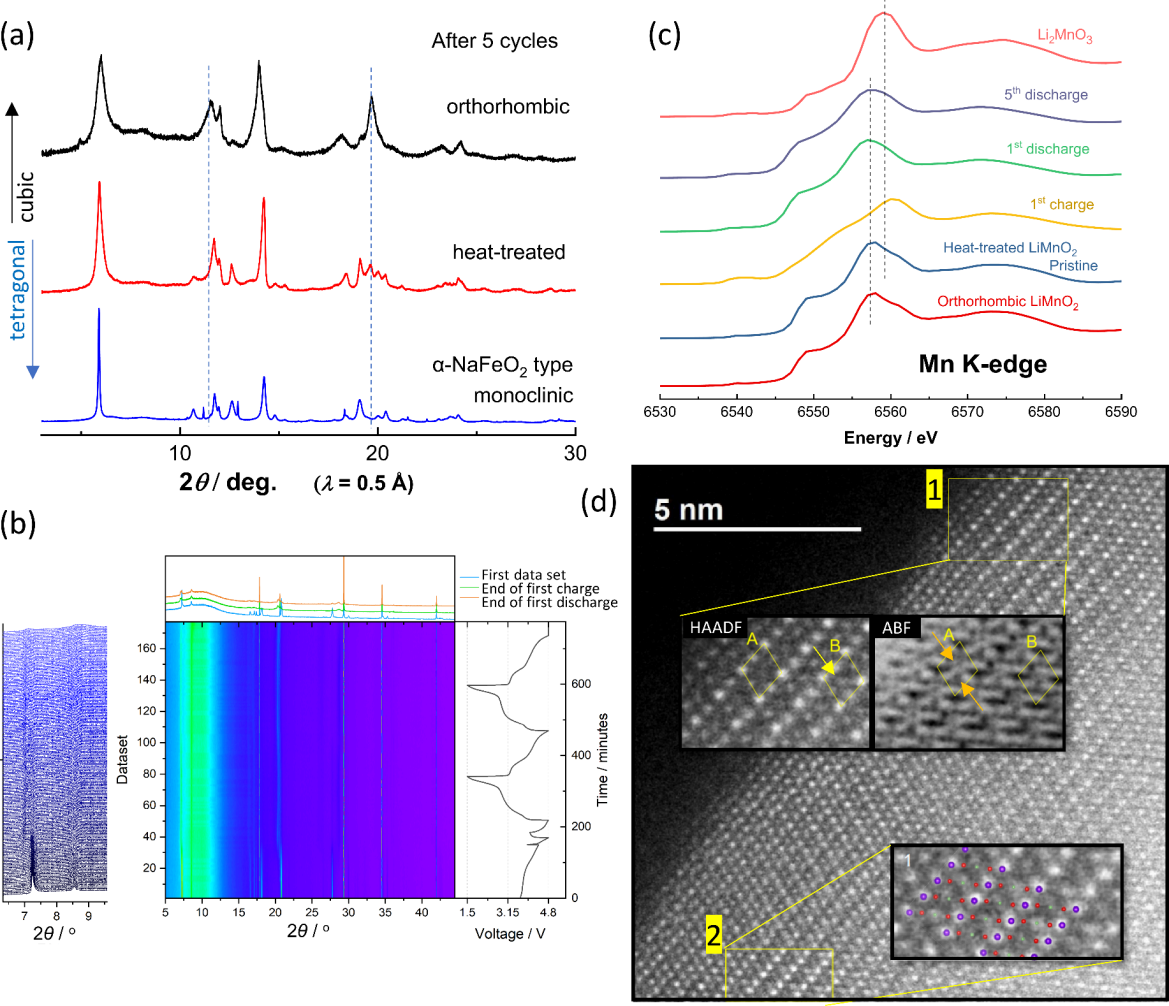
Structural characterization of different LiMnO_2_ polymorphs: (a) XRD patterns after 5 cycles. (b) Contour plot of *operando* XRD patterns, (c) X-ray absorption spectra, and (d) a high-resolution STEM image of heat-treated LiMnO_2_. The data of (d) was taken after 5 cycles. For (b), the 1st charge has a slight voltage drop around 150 min and to compensate and reach 4.8 V around 220 min the cell underwent the profile shown.

As determined above, electrochemical properties, especially aspects such as voltage hysteresis and electrode kinetics, are highly dependent on the cation arrangements and migration pathways in LiMnO_2_ with different crystal structures. Reaction mechanisms of these samples were examined using synchrotron XRD, and the structural evolution with electrochemical cycling is compared in Supporting Figure S11. For the orthorhombic LiMnO_2_ there is a clear structural transformation at the first discharge which is essentially complete by the fifth discharge and a new low crystallinity or nanosized phase is formed (Supporting Figure S11a). In contrast, minimal changes and a maintenance of crystallinity is shown for monoclinic LiMnO_2_ at the first and fifth discharge states (Supporting Figure S11c). However, it is also noted that certain reflections are sharper, e.g., at 2θ ∼ 19°, which may indicate the presence of a tetragonal phase such as Li_2_Mn_2_O_4_, a lithiated phase of cubic spinel LiMn_2_O_4_.^29^ The cubic symmetry for the spinel phase is lost by the enrichment of Jahn–Teller active ions (high-spin Mn^3+^), and the structural distortion caused by Jahn–Teller ions results in the stabilization of the tetragonal phase.^30^ Synchrotron XRD patterns of LiMnO_2_ samples after 5 cycles are compared in Figure 3a. Crystallinity of the samples is clearly different, and monoclinic LiMnO_2_ after 5 cycles shows the highest crystallinity. The lowest crystallinity is noted for orthorhombic LiMnO_2_ after 5 cycles, and peak positions are different compared with those of the sample derived from monoclinic LiMnO_2_. This phase (lowest crystallinity) cannot be assigned to tetragonal Li_2_Mn_2_O_4_, and this sample is regarded as a low crystallinity cubic spinel phase without tetragonal distortion (see Supporting Figure S12). This phase is also classified as a partial cation-ordered rocksalt oxide with cubic symmetry and will be discussed in a later section. Heat-treated LiMnO_2_ with monoclinic layered domains also changes into a lower crystallinity phase, but its crystallinity is clearly higher than that of the orthorhombic phase after 5 cycles. Moreover, the reflections are mainly assigned to the tetragonal spinel, probably with a partial presence of a cubic spinel phase.

Details of the phase transition for heat-treated LiMnO_2_ were further analyzed by an *operando* synchrotron XRD study (Figure 3b and Supporting Figure S13a and b). The ball-milled and heat-treated LiMnO_2_ electrode shows featured previously mentioned, unobservable (110) and higher intensity (021) reflections relative to the structural model of orthorhombic LiMnO_2_. Furthermore, the monoclinic phase was clearly observable as previously mentioned; see the (001) reflection in Supporting Figure S13c. The reflection at 2θ ∼ 7.3° is observed before charge corresponding to the orthorhombic (010) reflection shows a dramatic reduction in intensity with charge to 4.8 V but the reflection is still visible (see Supporting Figure S13c). In this process, this reflection splits into two, original orthorhombic and a secondary phase, and a change in the 2θ values are noted. This secondary phase is most likely an orthorhombic phase with a slightly expanded lattice by Li extraction, considering the smaller 2θ value. During discharge to 1.5 V, no change in the intensity or 2θ value for the orthorhombic (010) reflection is noted, but subtle changes in the 2θ value for the reflection associated with the secondary phase are noted. The presence of the orthorhombic reflection after the initial cycle is consistent with *ex-situ* XRD data (Supporting Figure S11). However, the orthorhombic phase almost disappears on the second charge process, indicating that the phase transition to the spinel-like phase is relatively fast for the heat-treated sample. The secondary phase remains relatively stable once formed and appears to slowly lose reflection intensity on the charging or 4.8 V potentiostatic hold steps. Similarly, the monoclinic phase shows a reduction in intensity during first charge and then remains relatively stable until the third charge and potentiostatic hold process, where the reflection noticeably broadens (Supporting Figure S13c). Furthermore, the *operando* XRD study reveals that the phase transition process for essentially all of the LiMnO_2_ phases predominantly occurs on charge, and this is clearly emphasized when the potential at 4.8 V is held (Supporting Figure S13c).

The formation of the spinel-like phase from the starting mixture of the orthorhombic and monoclinic in ball-milled and heat-treated LiMnO_2_ is rapid and occurs on the first charge process as shown in Supporting Figure S13d and e. By the end of first charge, the spinel-like reflections, *cf*. (200) as shown in Supporting Figure S13d, are clearly visible and very intense in the XRD patterns. These reflections are very broad indicating nanosized particles, estimated via the Scherrer equation on the spinel (220) reflection to be 86 nm on first charge and reducing marginally to 58 nm on third charge. Once formed, the spinel phase shows minimal changes with electrochemical cycling; see Supporting Figure S13f. This implies a very structurally stable phase upon cycling.

To examine the charge compensation processes which accompany these structural changes on electrochemical cycles, X-ray absorption spectroscopy (XAS) spectra was collected from pristine and cycled materials at the Mn K-edge region. For as-prepared samples, the profiles of Mn K-edge spectra are identical for both orthorhombic and heat-treated samples, as shown in Figure 3c, indicating that the samples contain trivalent Mn ions with a high-spin configuration (t_2g_^3^). After delithiation from heat-treated LiMnO_2_, a clear shift of the K-edge XAS spectrum to a higher energy region indicates the oxidation of Mn to a tetravalent state. Although its profile is slightly different from that of Li_2_MnO_3_ with tetravalent Mn ions, this trend originates from the fact that many vacant octahedral sites around Mn ions for the charged sample influences X-ray absorption processes by Mn ions.^31^ The energy of the K edge returns upon discharge, indicating reversible Li insertion-extraction processes coupled with cationic Mn^3+^/Mn^4+^ redox. Good reversibility is noted after 5 cycles, which is different from the observation for the Li-rich Mn-based system. Unstable oxygen redox is used for the Li-rich system, which often results in continuous reduction of Mn (Co) ions to lower oxidation states.^10,32^ The reversibility of Mn oxidation states is also consistent with stable cycling without voltage decay for heat-treated LiMnO_2_ (Figure 2d and Supporting Figure S8).

Changes in cation arrangements for heat-treated LiMnO_2_ with electrochemical cycling (after 5 cycles) were directly visualized by HAADF/ABF-STEM observation (Figure 3d and Supporting Figure S14a). Complex cation arrangements are noted after cycling. The sample contains nanosized domains that are about 5–20 nm in size with enriched domain boundaries, consistent with previous work.^33^ As shown in Supporting Figure S14a, “point a”, a clear cation arrangement related to a spinel-type host structure, as also highlighted in Figure 3d, it is found. In the ABF image, in which lighter ions are clearly visualized, the presence of ions is noted at tetrahedral sites (“point 1–A” in Figure 3d), and these ions are expected to be lighter Li ions in the spinel structure. When compared with heat-treated LiMnO_2_, smaller and nonuniform nanosized domains are found for orthorhombic LiMnO_2_ after cycling (Supporting Figure S14b), which is consistent with a previous finding.^19^ However, it is also difficult to conclude that all nanosized domains have spinel-type cation arrangements. In “point 2” in Figure 3d, and “point b” in Supporting Figure S14a, layered domains with a clear contrast in alternate octahedral layers are found. The presence of ions between two octahedral layers is also noted in the HAADF image, in which heavier ions are more emphasized. This phase is therefore regarded as a partially Mn ordered layered phase. Note that similar layered domains are more enriched for the sample derived from monoclinic layered LiMnO_2_ (Supporting Figure S14c). Additionally, in “point 1-B” in Figure 3d, and “point c” in Supporting Figure S14a, some domains show that all octahedral sites have uniform contrast, suggesting that Mn disordered rocksalt domains are also present.

### Factors Affecting the Phase Transition to a Spinel-Related Structure

**Figure 4:**
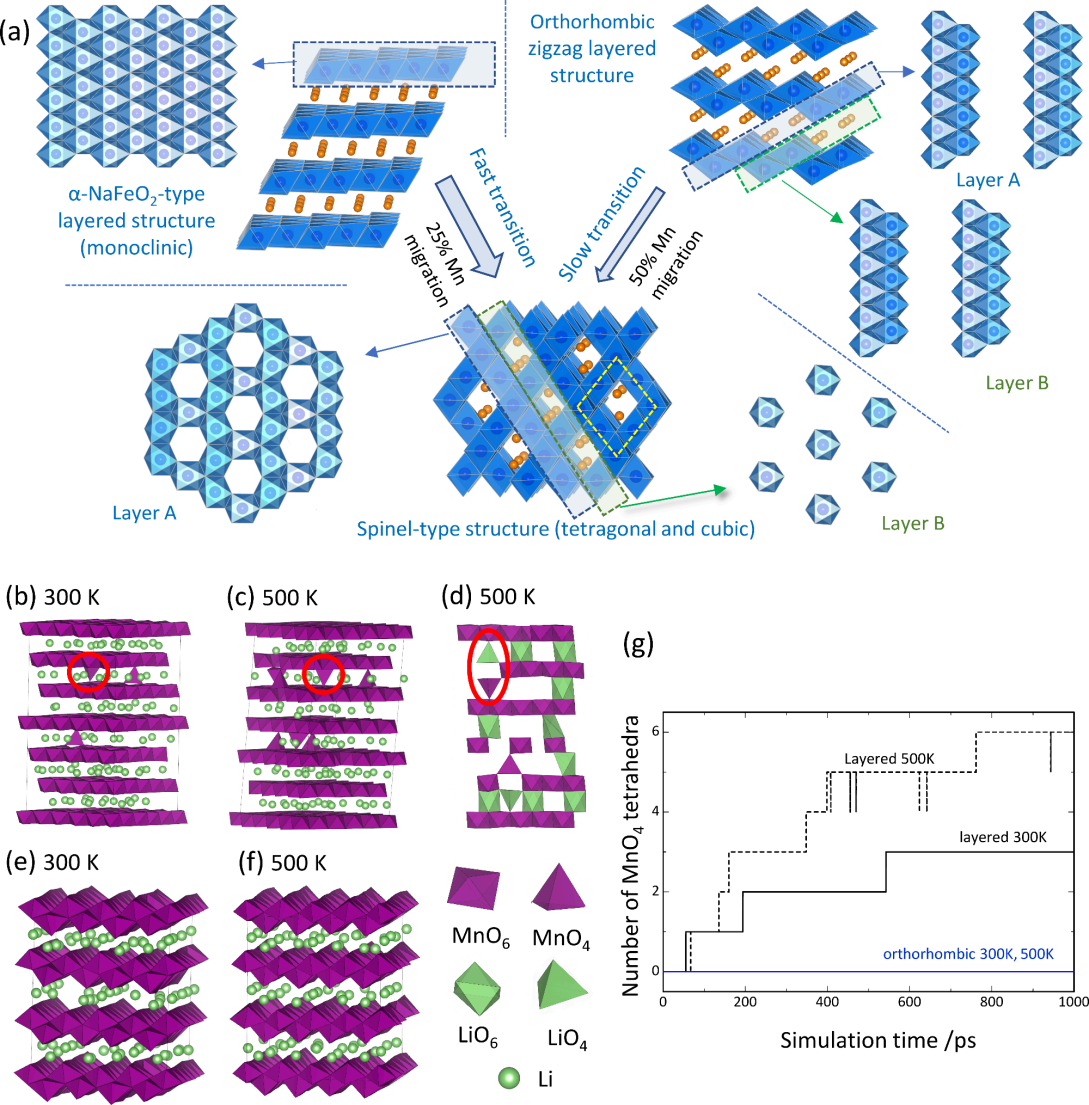
Phase evolution on electrochemical cycles for different LiMnO_2_ polymorphs: (a) Schematic illustrations for phase transition processes and computational study for phase transition for delithiated phases (also see Supporting Figure S1c), with (b)–(d) showing the monoclinic-derived Li_0.5_MnO_2_, (e)–(f) the orthorhombic-derived Li_0.5_MnO_2_ and (g) comparison of stability with molecular dynamics simulations.

The increase in reversible capacity on electrochemical cycling (Figure 2a) coupled with structural evolution studied by synchrotron XRD (Figure 3a) reveals that phase transition processes and their kinetics depend on the crystal structures in the as-prepared samples. Among the tested samples, α-NaFeO_2_-type monoclinic LiMnO_2_ shows the fastest phase transition with relatively higher crystallinity, whereas the slowest phase transition and the increase in reversible capacities for the initial several cycles are evidenced for orthorhombic LiMnO_2_. Heat-treated LiMnO_2_, with both domains, showed intermediate behavior. The kinetics of phase transition would be expected to be influenced by the cation distribution in the original phases. Note that both orthorhombic and monoclinic LiMnO_2_ have a common packing for oxide ions, i.e., cubic close-packed structure, and a difference is found for cation arrangements at octahedral sites. The spinel-type framework structure also features the same oxygen packing regime. Therefore, phase transitions from orthorhombic/monoclinic LiMnO_2_ to spinel Li_*x*_MnO_2_ are ideally achieved only by cation migration. If 1/4 of Mn ions in MnO_2_ slab in monoclinic LiMnO_2_ cooperatively migrate into adjacent octahedral sites in Li layers,^34^ a monoclinic layered to spinel phase transition occurs as shown in Figure 4a (also see Supporting Figure S1c). In contrast, the migration of 50% of Mn ions are required for the orthorhombic to spinel-like phase formation.^35^ This fact clearly influences the kinetics of phase transition, and therefore, the phase transition and lower crystallinity phase are only observed after cycling for orthorhombic LiMnO_2_.

To further study phase transition kinetics, molecular dynamics calculations were performed on Li_0.5_MnO_2_ derived from orthorhombic/monoclinic LiMnO_2_ using the Universal Neural Network Potential (UNNP). The UNNP learns using a deep neural network approach from the results of first-principles DFT calculations performed on various compositions and structures. This enables the computation of large-scale models while maintaining the accuracy of DFT calculations.^36^ The validity of this approach was evaluated by comparing the calculation results from both large-scale first-principles calculations and the UNNP. Supporting Figure S15 is a diagnostic plot for the energies and force components (in *x*, *y*, and *z* directions) acting on each ion obtained from both DFT and UNNP calculations. Because the coefficient of determination and the slopes are close to 1 for both energies and forces, UNNP sufficiently reproduces DFT calculations for the Li_0.5_MnO_2_ composition. Figures 4b–f shows snapshots of crystal structures after 1 ns of NPT-MD calculations for Li_0.5_MnO_2_, derived from monoclinic layered and orthorhombic LiMnO_2_. In the monoclinically derived Li_0.5_MnO_2_, three Mn ions in the octahedral sites migrate to the tetrahedral sites in the Li layer at 300 K (Figure 4b). At 500 K, the number of Mn ions located at the tetrahedral sites increases to six (Figure 4c). Moreover, a dumbbell configuration is observed, in which Li-vacancy-Mn atoms are arranged in the tetrahedral, octahedral, and tetrahedral sites in the direction perpendicular to the layer, shown in Figure 4d. This cation arrangement is consistent with the mechanism of Mn ion migration proposed by Reed et al. using first-principles DFT calculations.^37,38^ Multivalent ions are generally believed to have low diffusivity within oxide lattices, but divalent Mn ions formed by disproportionation reaction of Mn^3+^ move even at room temperature in conjunction with Li ions according to the literature.^37^ This behavior leads to the phase transition during electrochemical cycling; however, if the migration requires Mn^2+^ the propensity of such a transition would be limited to when Mn^2+^ is both high in concentration in the crystal structure and located near the planar defects discussed above. In contrast, no changes are observed in the host structure of Li_0.5_MnO_2_ derived from orthorhombic LiMnO_2_ at 300 and 500 K, confirming that the host structure is preserved (Figure 4e and f). This fact indicates that the diffusivity of Mn ions in the orthorhombic structure is qualitatively lower than that in the monoclinic structure and changes in the MnO_2_ structural framework are suppressed. Figure 4g shows changes in the number of Mn ions occupying the tetrahedral sites during the MD calculation process. As suggested in Figure 4e and f, there are no Mn ions moving to tetrahedral sites throughout the 1 ns simulation time in Li_0.5_MnO_2_ with an orthorhombic structure. On the other hand, the number of Mn ions occupying tetrahedral sites increases almost monotonically over time in Li_0.5_MnO_2_ with the monoclinic layered structure. (In the MD simulation at 500 K, some of Mn ions move back from tetrahedral sites temporarily to octahedral sites but quickly return to tetrahedral sites depending on the time scale of the calculation.) This movement of Mn does not show any sign of saturation, thus MD structure after 1 ns is not considered to be at equilibrium. Hence, it may continuously change from the layered structure to the more stable spinel phase. Very recently, Deng et al. conducted NPT-MD calculations on Li_0.5_MnO_2_ with an orthorhombic structure using a UNNP.^39^ At a high temperature of 1100 K, a phase transition from the orthorhombic structure to the spinel-type arrangement was found, further agreeing with our findings. By evaluating the changes in the XRD diffraction profile over time for the structures output by MD, the transition to the spinel phase was estimated to be around 0.8 ns at 1100 K. From these results, it is concluded that a phase transition is occurring due to a phenomenon where the constituent ions move simultaneously or cooperatively. As no movement of Mn ions is found in the MD calculations below 500 K within 1 ns in the orthorhombic structure, it further highlights that the phase transitions are relatively slow for the orthorhombic structure, correlating with the results shown in Figures 2 and 3.

### Practical Assessment of Nanostructured LiMnO_2_ for Battery Applications

**Figure 5:**
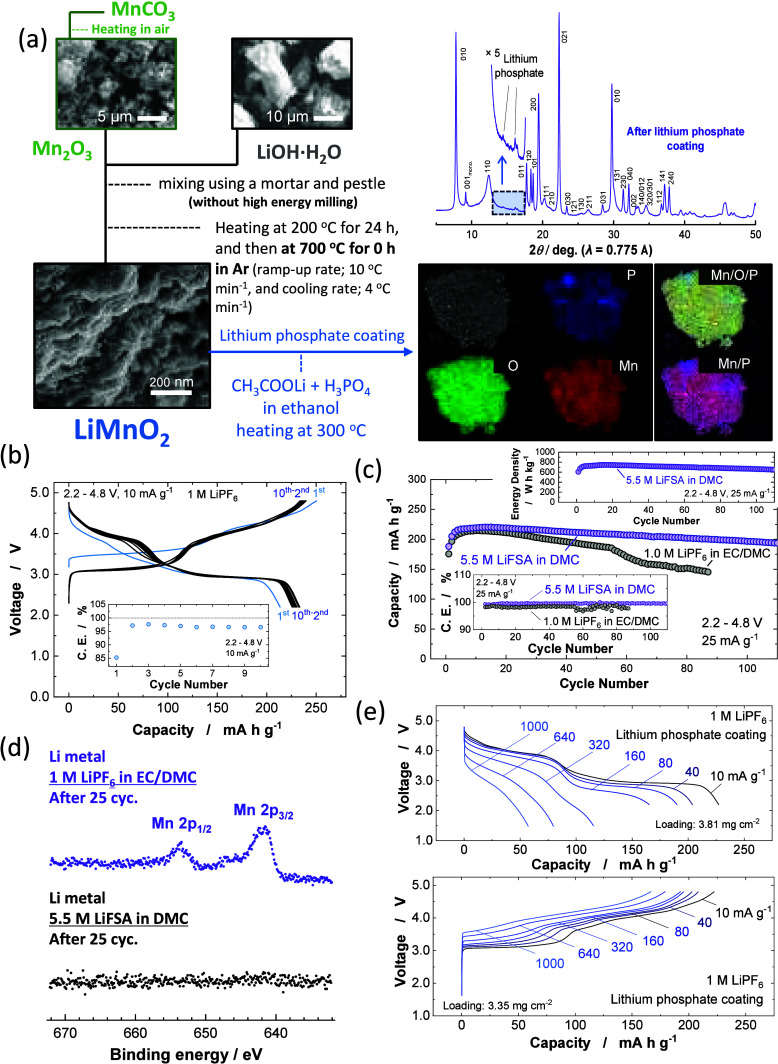
Direct synthesis and electrode performance of “nanostructured LiMnO_2_”: (a) A scheme of the synthesis of nanostructured LiMnO_2_ and lithium phosphate coating. Synchrotron XRD data and STEM/EDX data of lithium phosphate coated LiMnO_2_. (b) Galvanostatic charge/discharge curves of nanostructured LiMnO_2_ at a rate of 10 mA g^–1^, (c) capacity and energy density retention of lithium phosphate coated LiMnO_2_ in conventional electrolyte and highly concentrated electrolyte solutions at a rate of 25 mA g^–1^, (d) Mn 2p XPS spectra of metallic Li electrodes after cycling in different electrolyte solutions, and (e) discharge/charge rate capability of lithium phosphate coated LiMnO_2_ in the conventional electrolyte solution.

Detailed analysis for three LiMnO_2_ polymorphs reveals that the difference in the original crystal structures influences phase transition kinetics and phase crystallinity after electrochemical cycling, but no correlation with electrode reversibility and voltage hysteresis is noted. Nevertheless, as shown in Figure 1b and Supporting Figure S1, another important difference for these samples is found in grain sizes. Heat-treated LiMnO_2_ has a small particle size, <100 nm with good uniformity, which originates from the morphological character of the precursor, nanosized rocksalt LiMnO_2_ synthesized by high-energy milling. This fact also suggests that LiMnO_2_ with good electrode reversibility can be synthesized without high-energy milling and the precursor rocksalt LiMnO_2_ through particle size engineering. To test this hypothesis, we directly synthesized nanostructured LiMnO_2_ by using a simple calcination approach. Mn_2_O_3_ was thoroughly mixed with LiOH·H_2_O, a highly reactive precursor with a lower melting point compared with Li_2_CO_3_. The mixture of Mn_2_O_3_ and LiOH·H_2_O was pelletized and heated at 200 °C in an inert atmosphere to remove water. After water was removed, the temperature of the furnace was increased from 200 to 700 °C with a ramp-up rate of 10 °C min^–1^. After reaching the temperature to 700 °C, the furnace was immediately cooled to room temperature at a rate of 4 °C min^–1^. Note, the sample was heated inside Cu foil to avoid the oxidation and the detailed methodology has been described in the literature for similar materials.^40^ An XRD pattern and SEM image of the synthesized sample are shown in Supporting Figure S16a and b, respectively. As shown in Supporting Figure S16a, this sample is crystallographically similar to the sample derived from metastable rocksalt LiMnO_2_. Furthermore, its grain size is small (Figure 5a and Supporting Figure S16b), and moreover, the sample delivers a large reversible capacity, ∼230 mA h g^–1^ at a rate of 10 mA g^–1^ with 2.2 V discharge cutoff (Figure 5b). The surface area of the sample was measured to be 4.4 m^2^ g^–1^ by the BET method, which is comparable to the surface area (5.1 m^2^ g^–1^) observed for heat-treated LiMnO_2_ derived from the LiMnO_2_ rocksalt precursor. This alternative synthesis route is cost-effective and significantly simpler and produces a structure similar to that of heat-treated LiMnO_2_ with monoclinic and orthorhombic domains.

Practically, the dissolution of Mn ions into electrolyte solution is known as a significant problem for Mn-based electrode materials.^41^ Therefore, two methodologies were further applied; (1) surface coating by “phosphate” ions and (2) the use of a highly concentrated electrolyte solution. Lithium phosphate is less soluble in polar solvents and the elimination of free-solvent in highly concentrated electrolyte solutions results in the lower solubility of electrode materials.^13^ The coating of lithium phosphate has been conducted by using the methodology reported in literature.^42^ As shown in Figure 5a, after the coating by lithium phosphate, the presence of phosphate ions on the particle of LiMnO_2_ is evidenced by FE-SEM/EDX observation. A trace of crystalline lithium phosphate is also found by synchrotron XRD data (Figure 5a and Supporting Figure S16a). Although a similar initial capacity is observed for LiMnO_2_ before/after coating (Supporting Figure S16c), improved capacity retention is achieved for the coated sample (Supporting Information, Figure S16d) in conventional electrolytes. However, capacity is gradually lost with cycling to 60 cycles, and further degradation of the reversibility is observed after 60 cycles. This originates from the dissolution of the Mn ions from LiMnO_2_ and the accumulation of Mn ions on the metallic Li electrode. Electrolyte decomposition cannot be avoided when Mn ions are deposited on the surface of negative electrodes.^43^ Indeed, clear evidence of Mn ion deposition on the surface of metallic Li is noted by XPS (Figure 5d).

Electrode reversibility of LiMnO_2_ is significantly improved by using highly concentrated electrolyte solution with Li(NSO_2_F)_2_ (LiFSA) and dimethyl carbonate (DMC), and 5.5 M LiFSA/DMC.^44^ The maximum reversible capacity of ∼220 mA h g^–1^ at a rate of 25 mA g^–1^ after 10 cycles is found, and 90% of this capacity is retained even after 100 cycles (Figure 5c). The energy density reaches 730 Wh kg^–1^ at the 10th cycle, and 660 Wh kg^–1^ is retained at the 100th cycle. Improved Coulombic efficiency (99.5% on average) is also noted with 5.5 M LiFSA/DMC when compared with the conventional electrolyte (98.3% on average from 10 to 55 cycles). The electrochemical data of noncoated LiMnO_2_ with 5.5 M LiFSA/DMC is also shown in Supporting Figure S16e, and 83% capacity retention is achieved after 100 cycles. Note, Mn ions on the surface of metallic Li electrode cycled in 5.5 M LiFSA/DMC are not detected via XPS (Figure 5d), indicating that the dissolution of Mn ions is effectively suppressed with the highly concentrated electrolyte solution. Moreover, excellent quick charge capability is also found, Figure 5e, and approximately 75% of the capacity is recharged at a rate of 1000 mA g^–1^. These facts indicate the high practical potential of LiMnO_2_ coated with lithium phosphate. Further improvement of electrode reversibility is anticipated through mitigation of Mn dissolution on electrochemical cycling.

## Conclusions

Three different LiMnO_2_ polymorphs are synthesized, thermodynamically stable orthorhombic phase, metastable monoclinic layered phase derived from NaMnO_2_, and a phase with both orthorhombic and monoclinic domains derived from rocksalt LiMnO_2_. Electrode performance of these polymorphs has been systematically compared, and detailed analysis of differences in reaction mechanisms reveals that (1) the monoclinic layered domain effectively activates structural transition to the spinel-like phase, associated with structural similarity for monoclinic layered and spine-type structures, and (2) the surface area of particles influences reversible capacities and voltage hysteresis on electrochemical cycles. From these findings, nanostructured LiMnO_2_ with domain structures has been directly synthesized from LiOH·H_2_O and Mn_2_O_3_ without the use of metastable precursors. Moreover, nanostructured LiMnO_2_ shows a higher energy density, which is competitive with Ni-enriched electrode materials used for state-of-the-art electric vehicles. Voltage decay associated with oxygen loss, as observed for Li_2_MnO_3_-based electrode materials, cannot be observed. The sample also provides excellent fast charge ability, which is an essential character for electric vehicle applications. Although a remaining practical problem was found in Mn dissolution and capacity loss on electrochemical cycles, this problem is also effectively mitigated by the use of a highly concentrated electrolyte solution coupled with lithium phosphate coating. These findings contribute the development of high-energy, low-cost, and sustainable positive electrode made from abundant Mn sources without Ni/Co, which potentially realizes the sustainable energy society without the dependence on fossil fuel in the future.

## Experimental Methods

### Synthesis of Materials

Orthorhombic LiMnO_2_ was prepared from Li_2_CO_3_ (98.5%, Kanto Kagaku) and Mn_2_O_3_ at 900 °C for 12 h in an Ar atmosphere. Excess Li_2_CO_3_ (3%) was used to compensate for verbalized Li ions on heating. Mn_2_O_3_ was obtained by heating MnCO_3_ (Kishida Chemical Co., Ltd.) in air. Metastable cubic rocksalt LiMnO_2_ was prepared by mechanical milling using a planetary ball mill (PULVERISETTE 7; FRITSCH).^21^ Orthorhombic LiMnO_2_ was used as a precursor for the mechanical milling. LiMnO_2_ (1.5 g) was mixed using a zirconia pot (45 mL) and zirconia balls (15.5 g) at 600 rpm for 12 h. After being milled for 12 h, the mixture was taken out from the container and mixed with a mortar and pestle to ensure sample uniformity during the milling. The mixture was again milled using a zirconia pot and balls at 600 rpm for 12 h. This process was repeated three times, and the mixture was milled for 36 h in total. Monoclinic layered LiMnO_2_ was prepared by ion-exchange from NaMnO_2_. NaMnO_2_ was added into LiBr dissolved in hexanol, and then heated at 160 °C for 96 h.^45^ After heating in hexanol, the ion-exchanged sample was filtered and dried. NaMnO_2_ was synthesized from Na_2_CO_3_ (99.5%; Wako Pure Chemical Industries, Ltd.) and Mn_2_O_3_ at 800 °C for 8 h in an Ar atmosphere. Heat-treated LiMnO_2_ was obtained by heat-treatment of cubic rocksalt LiMnO_2_ at 700 °C for 2 h in Ar atmosphere. The synthesized LiMnO_2_ polymorphs were stored in a desiccator in air with silica gel.

### Electrochemical Evaluations

The electrode performance of orthorhombic LiMnO_2_, cubic rocksalt LiMnO_2_, and monoclinic layered LiMnO_2_ was examined for the carbon composite samples prepared by ball milling. The samples were mixed with acetylene black, AB (HS-100; Denka Co., Ltd., LiMnO_2_:AB = 90:10 wt %) by using the planetary ball mill at 300 rpm for 12 h with the zirconia container and balls. Composite positive electrodes, comprising 76.5 wt % LiMnO_2_, 13.5 wt % AB, and 10 wt % poly(vinylidene fluoride), PVdF (KF 1100; Kureha Co. Ltd.), were pasted on aluminum foil as a current collector. Electrode performance of heat-treated LiMnO_2_ was evaluated without the preparation of a carbon composited sample, and a mixture of 80.0 wt % LiMnO_2_, 10.0 wt % AB, and 10.0 wt % PVdF was pasted on aluminum foil. Typical thickness of the composite electrode is 60 ± 10 μm, and the loading of active materials ranges from 3–4 mg cm^–2^. The composite electrode was evaluated without calendaring.

The electrodes were dried at 80 °C for 2 h in vacuum and then heated at 120 °C for 2 h. Metallic lithium (Honjo Metal Co., Ltd.) with a 250 μm thickness was used as a negative electrode. The electrolyte solution used was 1.0 mol dm^–3^ LiPF_6_ dissolved in ethylene carbonate (EC): dimethyl carbonate (DMC) with a volume ratio of 3:7, battery grade; Kishida Chemical Corp., Ltd.), and 300 μL of electrolyte solution was added for each electrochemical cell. Conventional polyolefin separator was used as the separator. LiN(SO_2_F)_2_ (LiFSA) dissolved in DMC was also used (LiFSA: DMC = 1:1.1 in molar ratio, battery grade; Kishida Chemical Corp., Ltd.) with aramid-coated polyolefin separator.^46,47^ Two-electrode cells (TJ-AC; Tomcell Japan) were assembled in an Ar-filled glovebox. An Al cell was used for a positive electrode side to avoid corrosion of cells made of stainless steel.^48^ The cells were cycled at a rate of 10 or 25 mA g^–1^ at room temperature.

### Lithium Phosphate Coating

LiMnO_2_ (0.5 g) was added into ethanol (10 mL), and then lithium acetate (Wako Pure Chemical Industries, Ltd.) ethanol solution (275 μL, 0.047 mol L^–1^) was added. Phosphoric acid (Wako Pure Chemical Industries, Ltd.) ethanol solution (122 μL, 0.060 mol L^–1^) was further added under stirring. The mixed solution was dried in vacuum, and the obtained powder was heated at 300 °C for 5 h in air.

### Material Characterization

Phase purity and crystal structures of the obtained samples were examined by using an X-ray diffractometer (D2 PHASER, Bruker) equipped with a high-speed one-dimensional detector. Nonmonochromatized Cu Kα radiation was utilized as an X-ray source with a nickel filter. Structural analysis was conducted using RIETAN-FP software.^49^ The reaction mechanisms of the electrode materials during cycling were examined by *ex-situ* SXRD (SPring-8, BL19B2) and hard X-ray absorption spectroscopy (Photon Factory, BL-9C) at the V K-edge. Composite electrode materials for these measurements were extracted from two-electrode cells after cycling at a rate of 10 mA g^–1^, and then rinsed with dimethyl carbonate. After being dried, the samples were packed into a capillary tube and sealed in a water-resistant polymer film in an argon-filled glovebox. The hard X-ray absorption spectra were collected with a silicon monochromator in the transmission mode. The intensity of the incident and transmitted X-rays was measured using an ionization chamber at room temperature. Normalization of the XAS spectra was conducted using the program code IFEFFIT.^50^ The background was determined by using a cubic spline procedure. Synchrotron X-ray diffraction study was also performed at the BL5S2 of Aichi Synchrotron Radiation Center.

*Operando* SXRD data were also collected using the Powder Diffraction Beamline at the Australian Synchrotron,^51^ using a wavelength λ = 0.727657(7) Å determined using a National Institute of Standards and Technology (NIST) 660b LaB_6_ standard reference material. Samples were cycled in Li half-cells by using CR2032 coin cell casings. A 3 mm hole was drilled through the cell casings and sealed using Kapton tape to allow for transmission of the incident beam through the sample. Diffraction data were collected in 6 min intervals while the cell was cycled from 1.5 to 4.8 V and this process was repeated for 2.5 cycles.

X-ray total scattering measurements was performed with an incident X-ray energy of *E* = 61.4 keV at the BL04B2 beamline in SPring-8, Japan, to study the local structure by pair distribution function (PDF) analysis. Hybrid detectors of Ge and CdTe were employed for the data collection of total structure factor *S*(*Q*). The reduced PDF *G*(*r*) was obtained by the conventional Fourier transform of *S*(*Q*).^52^

Scanning transmission electron microscopy (STEM) was conducted using a JEOL JEM-ARM200F with a CEOS CESCOR STEM Cs corrector (spherical aberration corrector) operated at an acceleration voltage of 200 kV. Details for experimental setup, including specimen preparation, are found in literature.^53^ Particle morphologies of the samples were observed using a scanning electron microscope (SEM; SU8010, Hitachi High-Technologies).

X-ray photoelectron spectroscopy (XPS) analysis of the metallic Li electrode was conducted with a PHI Quantera SXM spectrometer (Ulvac-phi). Electrochemical cells after 25 cycles were disassembled in the Ar-filled glovebox. Metallic Li electrodes obtained from the cells were rinsed with DMC and dried under vacuum, and then, the samples were transferred into the XPS instrument using an airtight transfer vessel without exposure to air. XPS measurements were conducted with an X-ray source (Al Kα) under a base pressure of 6.7 × 10^–8^ Pa.

The Brunauer–Emmett–Teller (BET) specific surface area of the samples was measured at 77 K on a micromeritics surface area and porosity analyzer (BELSORP MINI X; Microtrac MRB).

### Theoretical Analysis

Molecular dynamics calculations were performed using the Universal Neural Network Potential (UNNP). In this study, version 4.0.0 of the Preferred Potential (PFP) potential set implemented in the Matlantis software was used for the calculation.^54^ The validity of UNNP was evaluated by comparing the calculation results evaluated with first-principles calculations and UNNP. The models used for accuracy comparison were the structures of Li_18_Mn_36_O_72_ with layered and orthorhombic ordering. The structures were relaxed using first-principles calculations (DFT), and then distortions were introduced by randomly displacing the constituent atoms to create 50 structures for each structure (a total of 100 distorted structures are made). In detail, models were created by randomly displacing lattice constants by <0.1 Å, lattice angles by <2 degrees, and atomic positions within 0.05 Å. Energy calculations were performed on these models using both DFT and UNNP. First-principles calculations were performed using the Vienna ab initio simulation package (VASP)^55,56^ with the projector augmented-wave method (PAW).^57^ The Perdew–Burke–Ernzerhof functional (revised for solids) generalized gradient approximation (PBEsol-GGA) were used as the exchange-correlation functional.^58,59^ Also, the DFT+U method was used, setting the U value for Mn at 3.9 eV, referencing the literature.^60^ Molecular dynamics (MD) simulations were conducted on these models under isothermal–isobaric ensemble (NPT) conditions for 1 ns (10^6^ steps). In these MD simulations, superlattice models Li_75_Mn_150_O_300_ and Li_60_Mn_120_O_240_ for layered and orthorhombic ordering structures were created by scaling 5 × 5 × 2 times and 5 × 4 × 3 times, respectively.

## Supporting Information Available

SEM images of different LiMnO_2_ polymorphs, highlighted XRD patterns of orthorhombic and heat-treated LiMnO_2_, comparison of oxygen packing of orthorhombic and monoclinic layered LiMnO_2_, energy difference of LiMnO_2_ polymorphs obtained by theoretical calculations, STEM images of heat-treated LiMnO_2_, simulated XRD patterns with stacking fault analysis, structure factors and X-ray PDFs of different LiMnO_2_ polymorphs obtained by high-energy X-ray diffraction, charge/discharge curves of orthorhombic LiMnO_2_ before/after the preparation of carbon composited sample, charge/discharge curves and differential capacity plots of the as-prepared sample for monoclinic layered LiMnO_2_, cyclability of different LiMnO_2_ polymorphs at a rate of 10 mA g^–1^, comparison of electrode performance of heat-treated LiMnO_2_ and Li_1.2_Co_0.13_Ni_0.13_Mn_0.54_O_2_, comparison of quasi-open circuit voltage for heat-treated LiMnO_2_ (5th and 10th cycles), charge/discharge curves of LiNi_0.835_Co_0.15_Al_0.015_O_2_, structural evolution of different LiMnO_2_ polymorphs, simulated XRD patterns of cubic and tetragonal Li_2_Mn_2_O_4_ (Li_1+*x*_Mn_2_O_4_), selected Rietveld-refined fits of structural models to the *operando* XRD data for heat-treated LiMnO_2_, selected XRD patterns from the *operando* XRD experiment, *operando* XRD pattern overlaid, STEM images of heat-treated LiMnO_2_ after cycle test, diagnostic plots of energies and forces obtained by DFT and UNNP calculations, characterization and electrochemistry of nanostructured LiMnO_2_ (PDF)
