# Supplementary material for: A Practical and Sustainable Ni/Co-Free High-Energy Electrode Material: Nanostructured LiMnO2
Source: ACS Cent Sci. 2024 Aug 26;10(9):1718–32. doi: 10.1021/acscentsci.4c00578 (PMC11428382; doi:10.1021/acscentsci.4c00578)
Supplement: Supplementary file 1 — oc4c00578_si_001.pdf [file oc4c00578_si_001.pdf]

Supporting Information for

A Practical and Sustainable Ni/Co-free High-Energy Electrode Material: Nanostructured $LiMnO_2$

Miyaoka Yuka,[1†] Takahito Sato,[2†] Yuna Oguro,[1] Sayaka Kondo,[3] Koki Nakano,[3] Masanobu Nakayama,[3] Yosuke Ugata,[1, 4] Damian Goonetilleke,[5#] Neeraj Sharma,[5] Alexey M. Glushenkov,[6] Satoshi Hiroi,[7] Koji Ohara,[7] Koji Takada,[8] Yasuhiro Fujii,[8] and Naoaki Yabuuchi[1, 4*]

[1]Department of Chemistry and Life Science, Yokohama National University, 79-5 Tokiwadai, Hodogaya-ku, Yokohama, Kanagawa 240-8501, Japan

[2]Department of Applied Chemistry, Tokyo Denki University, 5 Senju Asahi-Cho, Adachi, Tokyo 120-8551, Japan

[3]Frontier Research Institute for Materials Science (FRIMS), Nagoya Institute of Technology, Gokiso-cho, Showa-ku, Nagoya, Aichi 466-8555, Japan

[4]Advanced Chemical Energy Research Center, Institute of Advanced Sciences, Yokohama National University, Yokohama, Japan

[5]School of Chemistry, University of New South Wales, Sydney, NSW 2052, Australia

[6]Research School of Chemistry, The Australian National University, Canberra ACT 2600, Australia

[7]Faculty of Materials for Energy, Shimane University, Matsue, Shimane 690-8504, Japan

[8]Tosoh Corporation, 4560 Kaisei-cho, Shunan-Shi, Yamaguchi 746-8501, Japan

[#]Present address: Corporate Research and Development, Umicore, Olen, Belgium

† These authors contributed equally to this work.

*Corresponding author, E-mail: yabuuchi-naoaki-pw@ynu.ac.jp

Supporting Figures

(a)

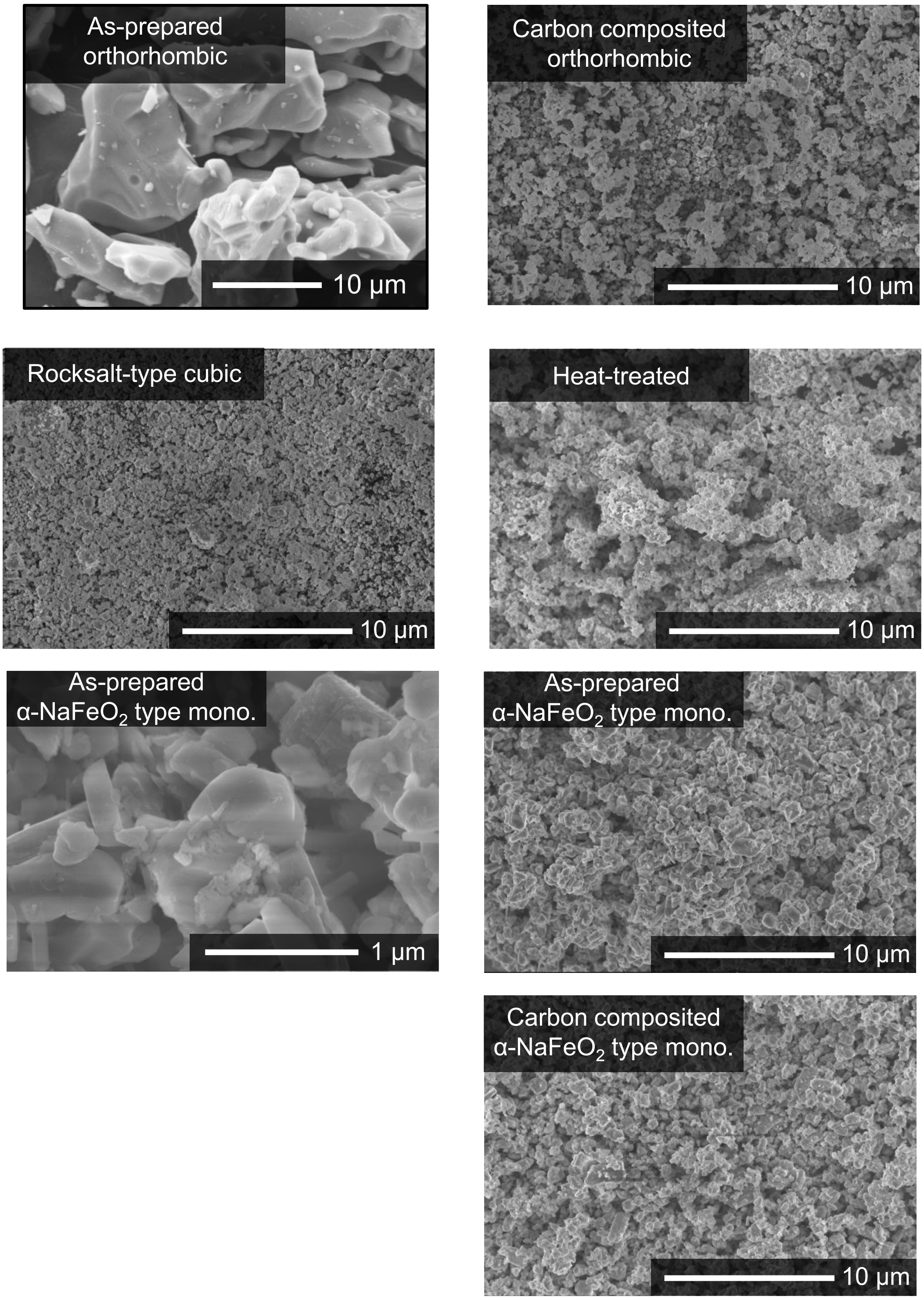

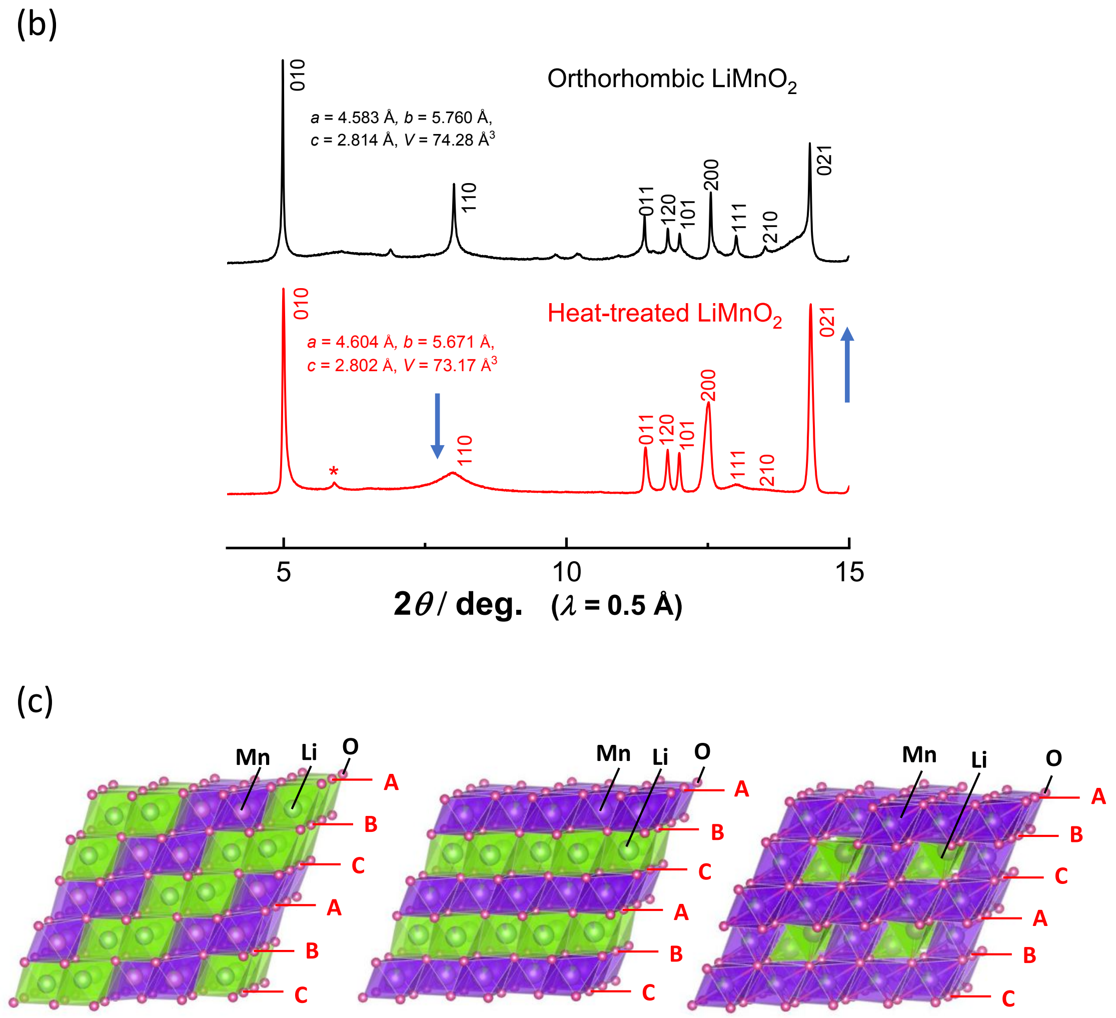

Figure S1. (a) SEM images of different $LiMnO_2$ polymorphs, (b) selected 2θ regions of the XRD patterns of orthorhombic and heat-treated $LiMnO_2$, arrows indicate the relative intensities of the reflections mentioned in the text, and (c) comparison of oxygen packing of orthorhombic (left) and monoclinic layered (middle) LiMnO2 with a spinel-type structure (right). Oxygen packing manners for orthorhombic and monoclinic phases are the same for each other, but cation distributions are different for both phases. In addition, the spinel phase is easily derived from the monoclinic layered phase as described in **Figure 4a**.

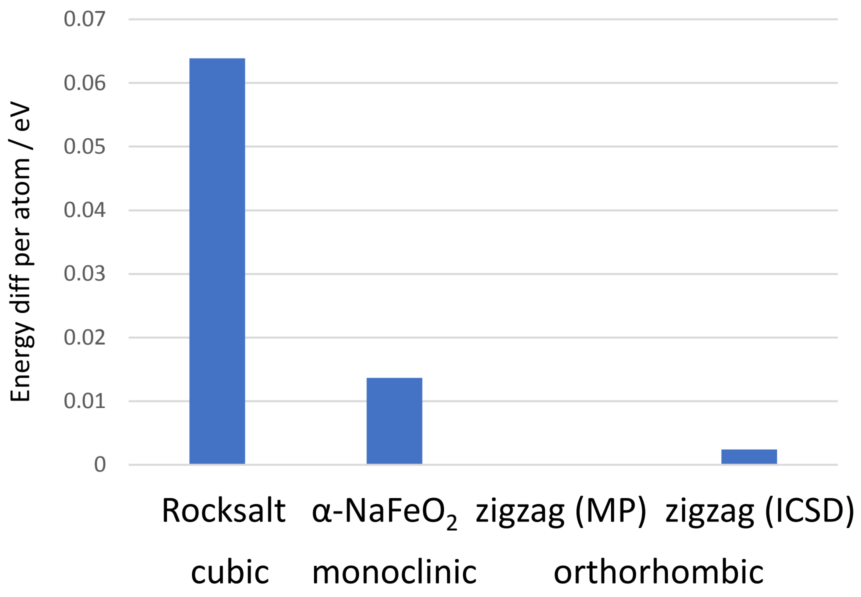

Figure S2. Energy difference of $LiMnO_2$ polymorphs obtained by theoretical calculations. The structural models of orthorhombic (zigzag) $LiMnO_2$ were taken from The Materials Project (MP) database and ICSD database. Slightly lower formation energy is obtained for the model in MP database.

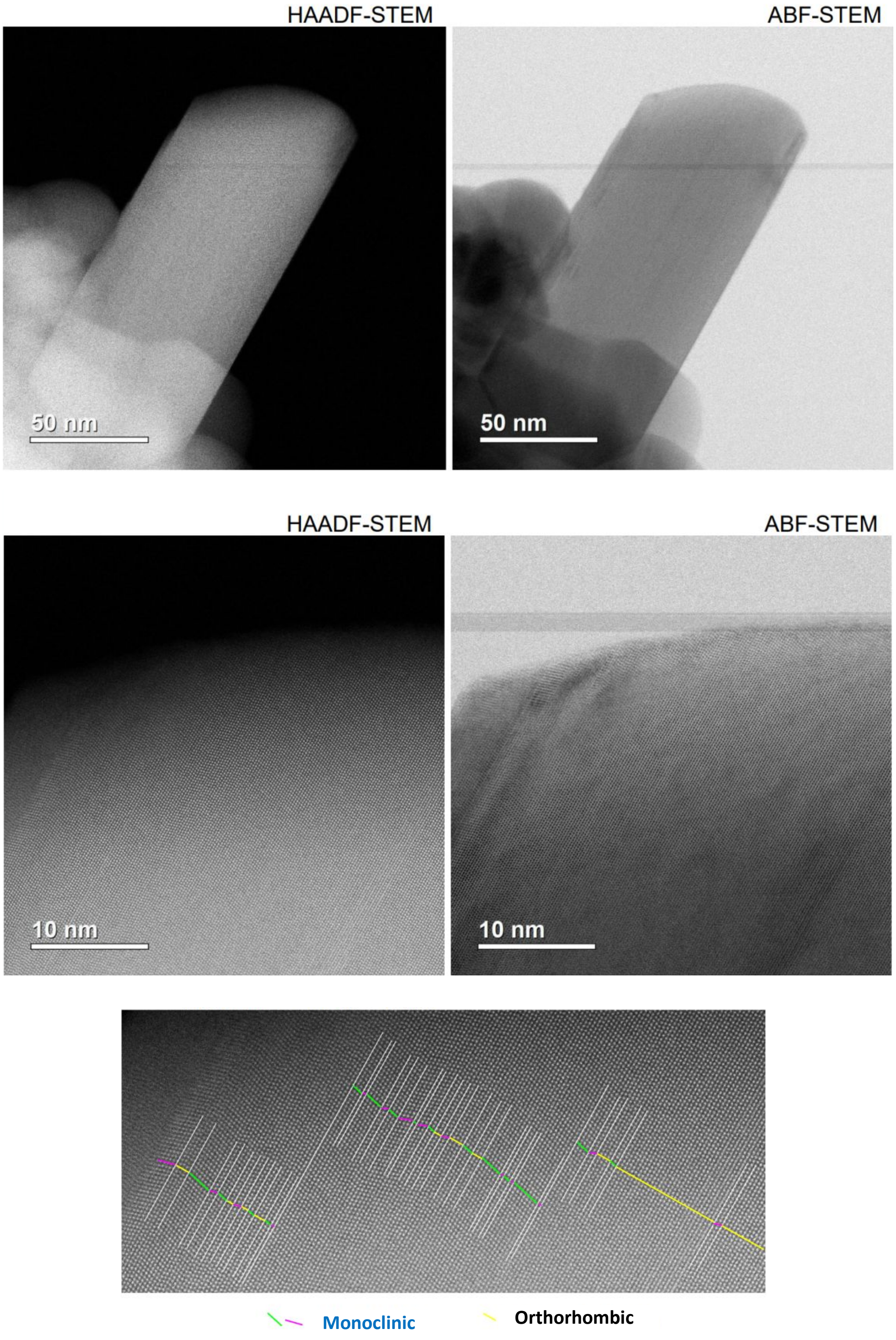

Figure S3. STEM images of heat-treated $LiMnO_2$. Planer stacking faults in the sample are also visualized.

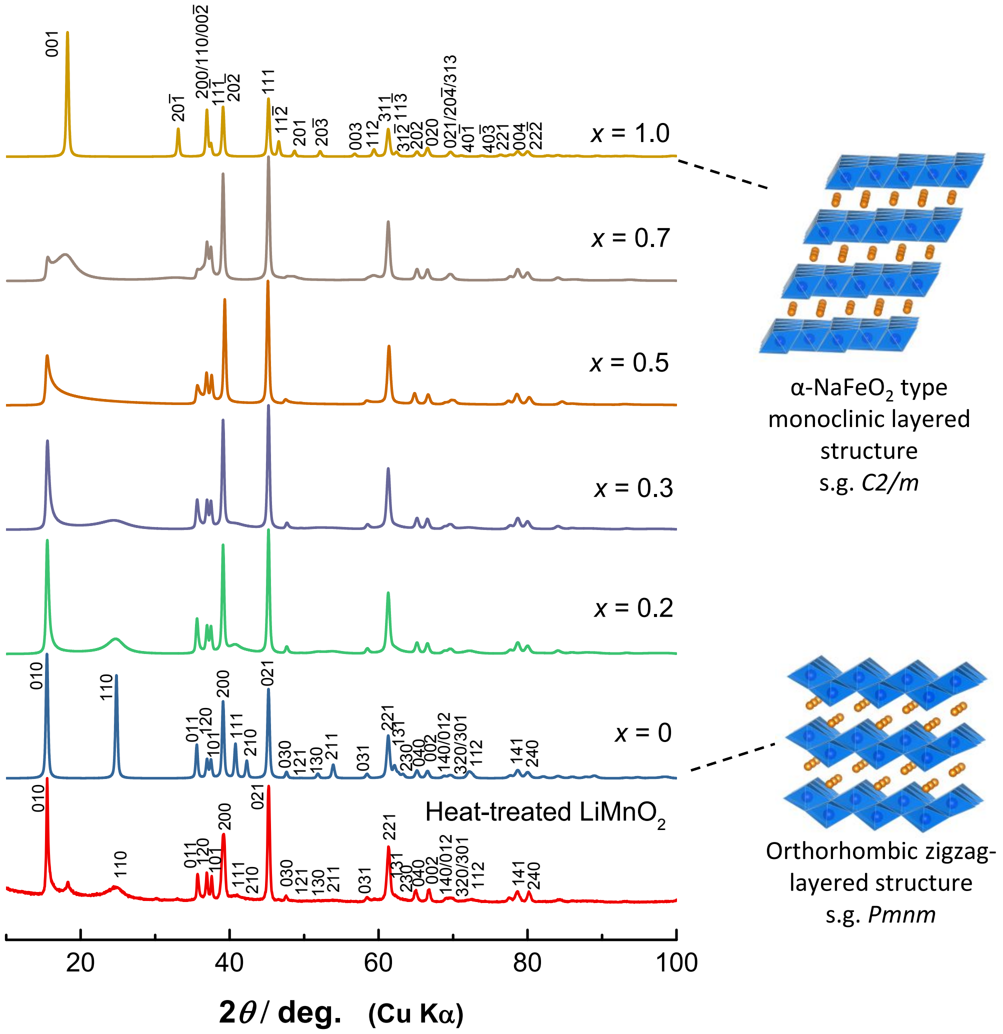

Figure S4. Simulated XRD patterns with stacking faults utilizing the DIFFaX program. Fractions of monoclinic layered and orthorhombic zigzag layered domains are changed; $x = 1.0$ corresponds to 100% monoclinic layered domain and $x = 0$ corresponds to 100% orthorhombic zig-zag layered domain. The model proposed by Croguennec *et al.*[1] was applied for the analysis by using DIFFaX program.[2]

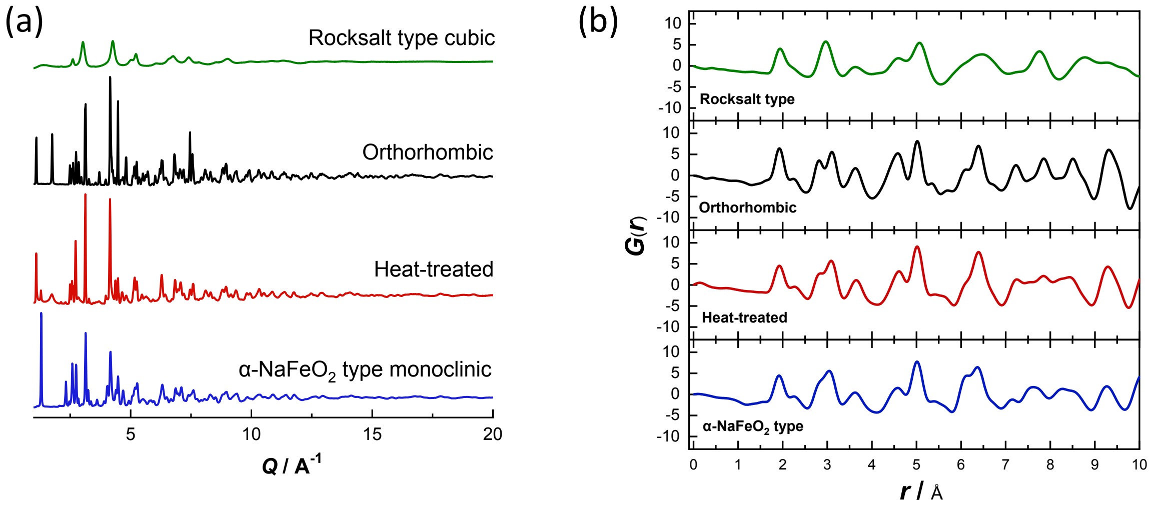

Figure S5. Structure factors of different $LiMnO_2$ polymorphs obtained by high-energy X-ray diffraction (E = 61.4 keV), from which (b) X-ray PDFs were obtained.

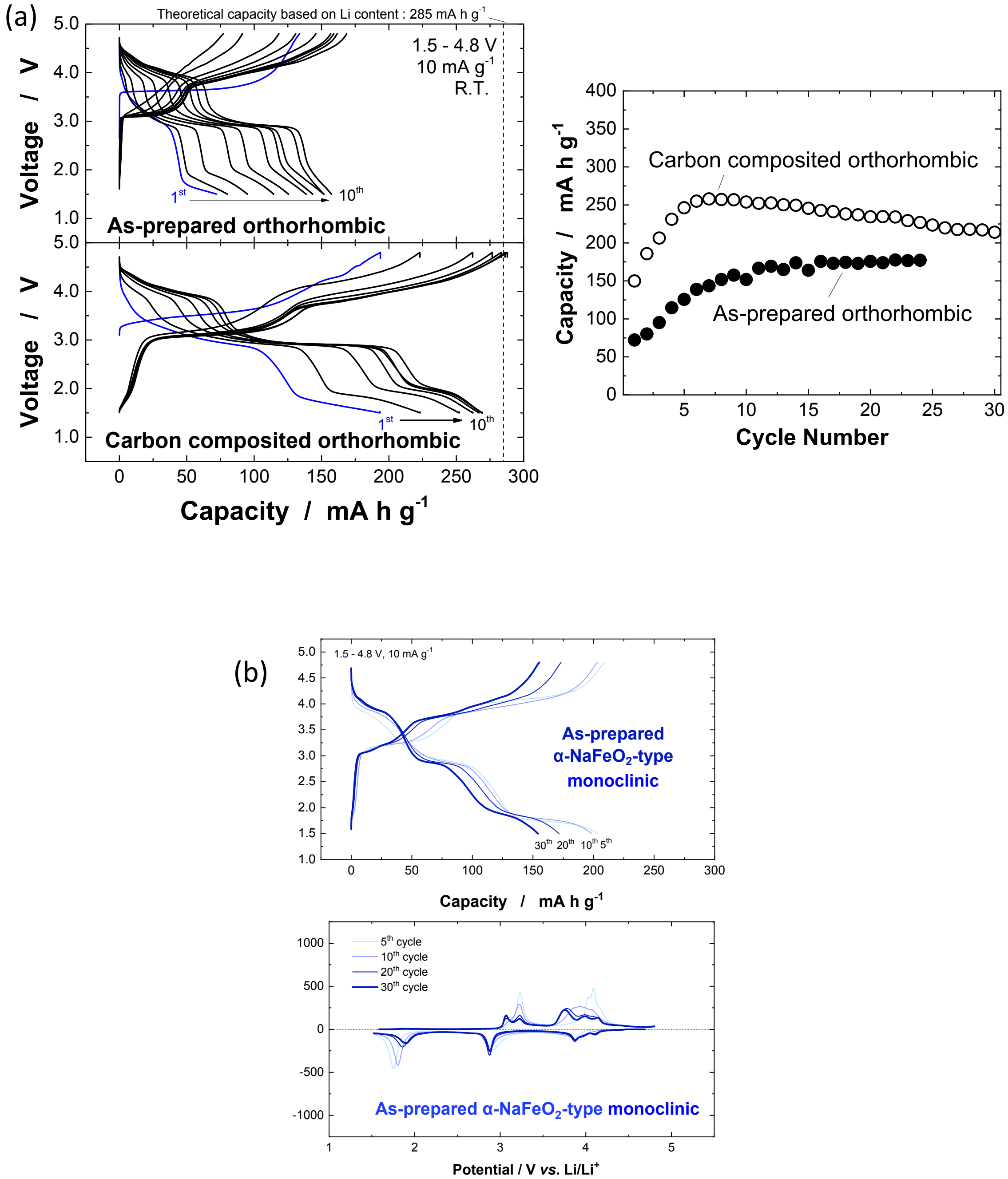

Figure S6. (a) Charge/discharge curves of orthorhombic $LiMnO_2$ before/after the preparation of carbon composited sample. The discharge voltage plateau at 2 V gradually disappears for the as-prepared orthorhombic sample, presumably associated with inferior discharge electrode kinetics. Better reversibility is evidenced for the carbon composited orthorhombic sample, but the gradual loss

of the 2 V plateau region, similar to the as-prepared sample, is observed on continuous cycles (**Figure 2c**). (b) Charge/discharge curves and differential capacity plots of the as-prepared sample for monoclinic layered $LiMnO_2$.

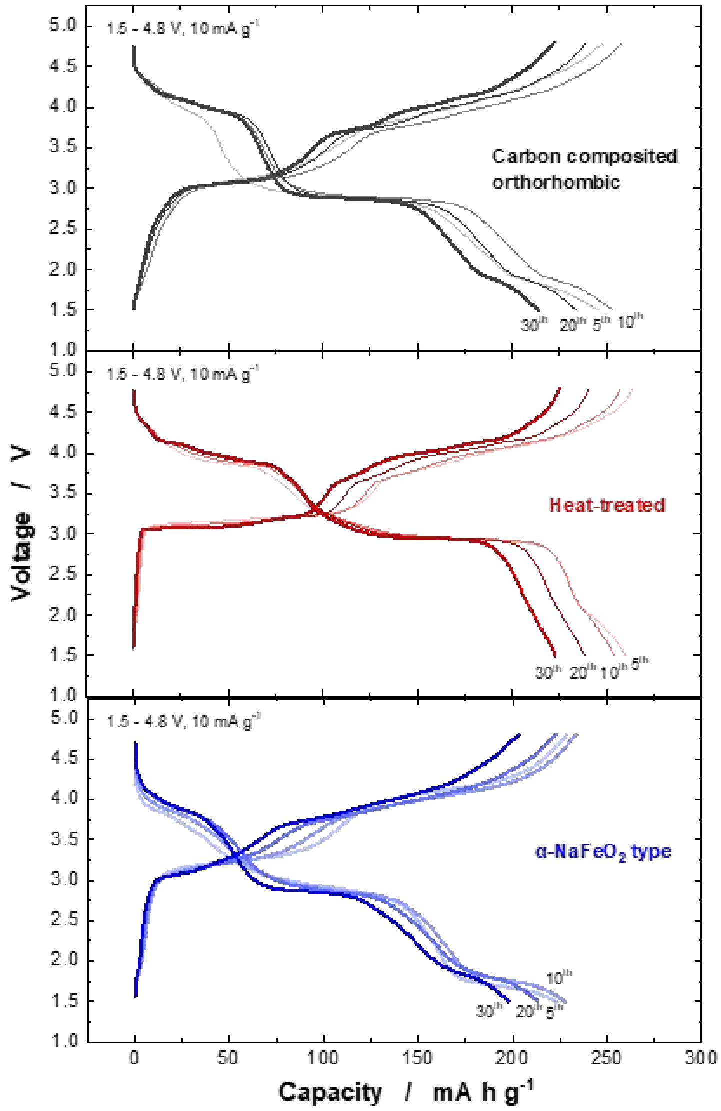

Figure S7. Cyclability of different $LiMnO_2$ polymorphs at a rate of 10 mA g^{-1}.

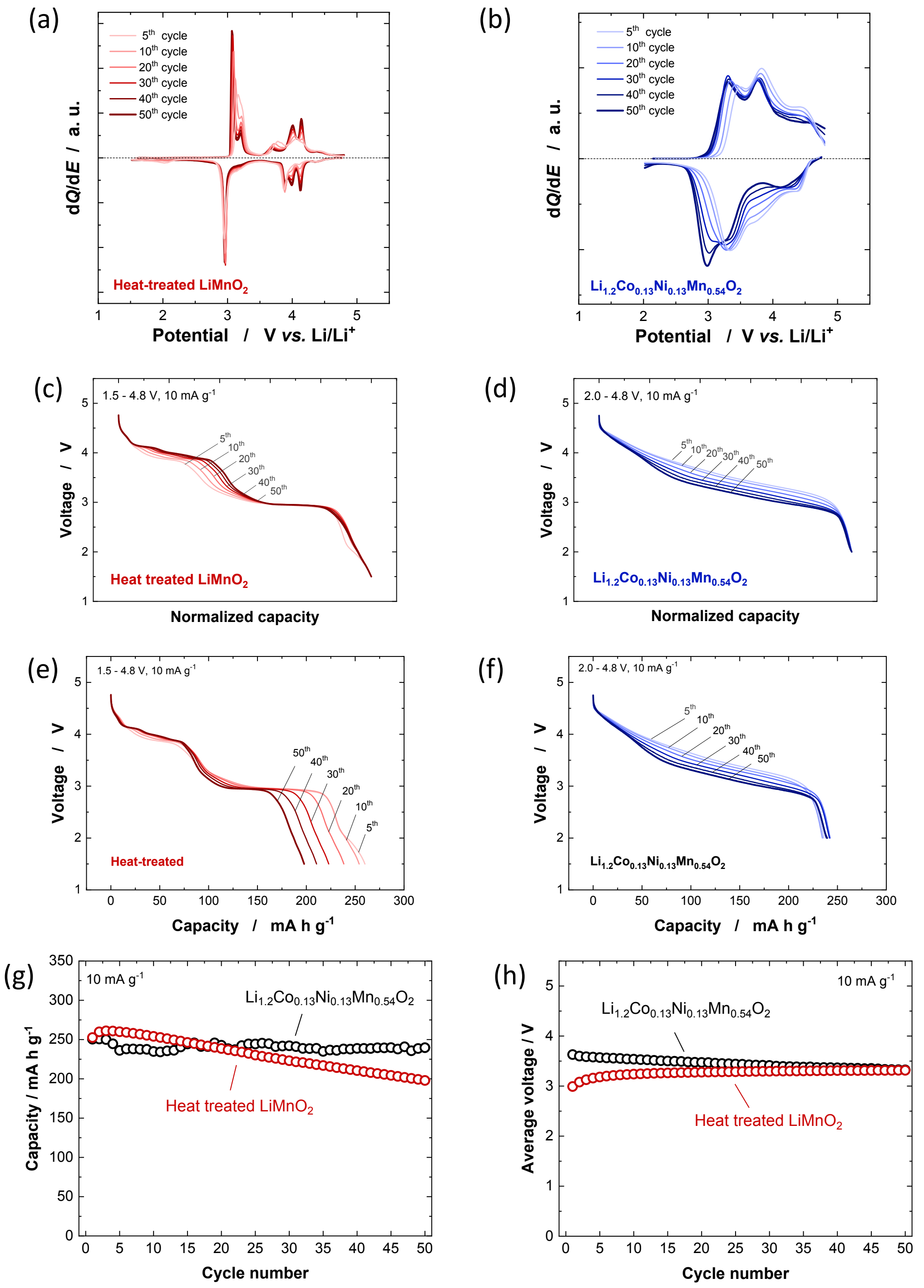

Figure S8. Comparison of electrode performance of heat-treated $LiMnO_2$ and $Li_{1.2}Co_{0.13}Ni_{0.13}Mn_{0.54}O_2$; (a, b) differential capacity plots, (c, d) normalized discharge curves, (e, f) discharge curves, (g) capacity retention, and (h) average discharge voltage.

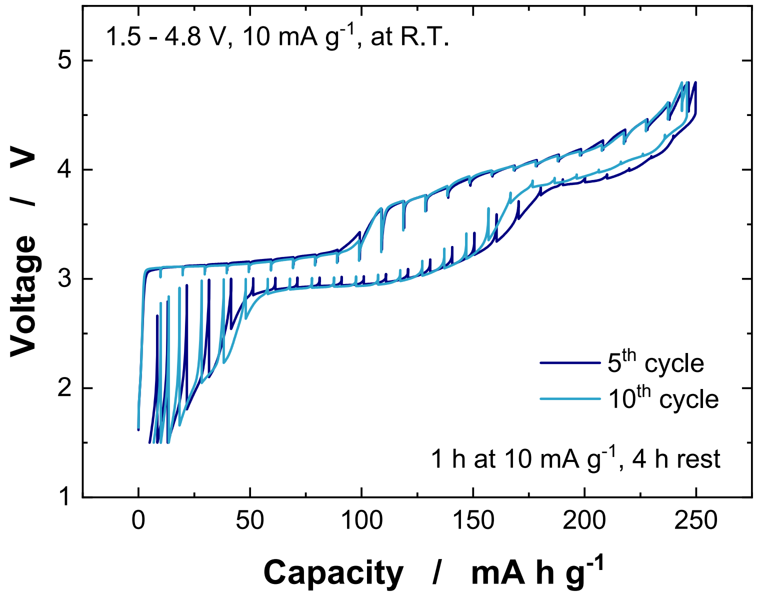

Figure S9. Comparison of quasi-open circuit voltage for heat-treated $LiMnO_2$ (5th and 10th cycles).

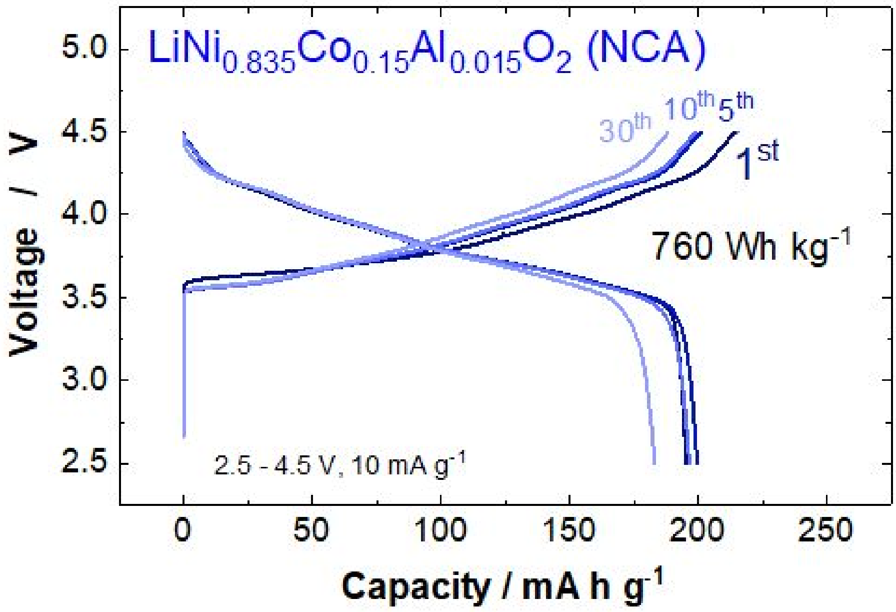

Figure S10. Charge/discharge curves of $LiNi_{0.835}Co_{0.15}Al_{0.015}O_2$ in the voltage range of 2.5 and 4.5 V at a rate of 10 mA g^{-1}. Energy density reaches ~760 Wh kg^{-1} based on the active material mass.

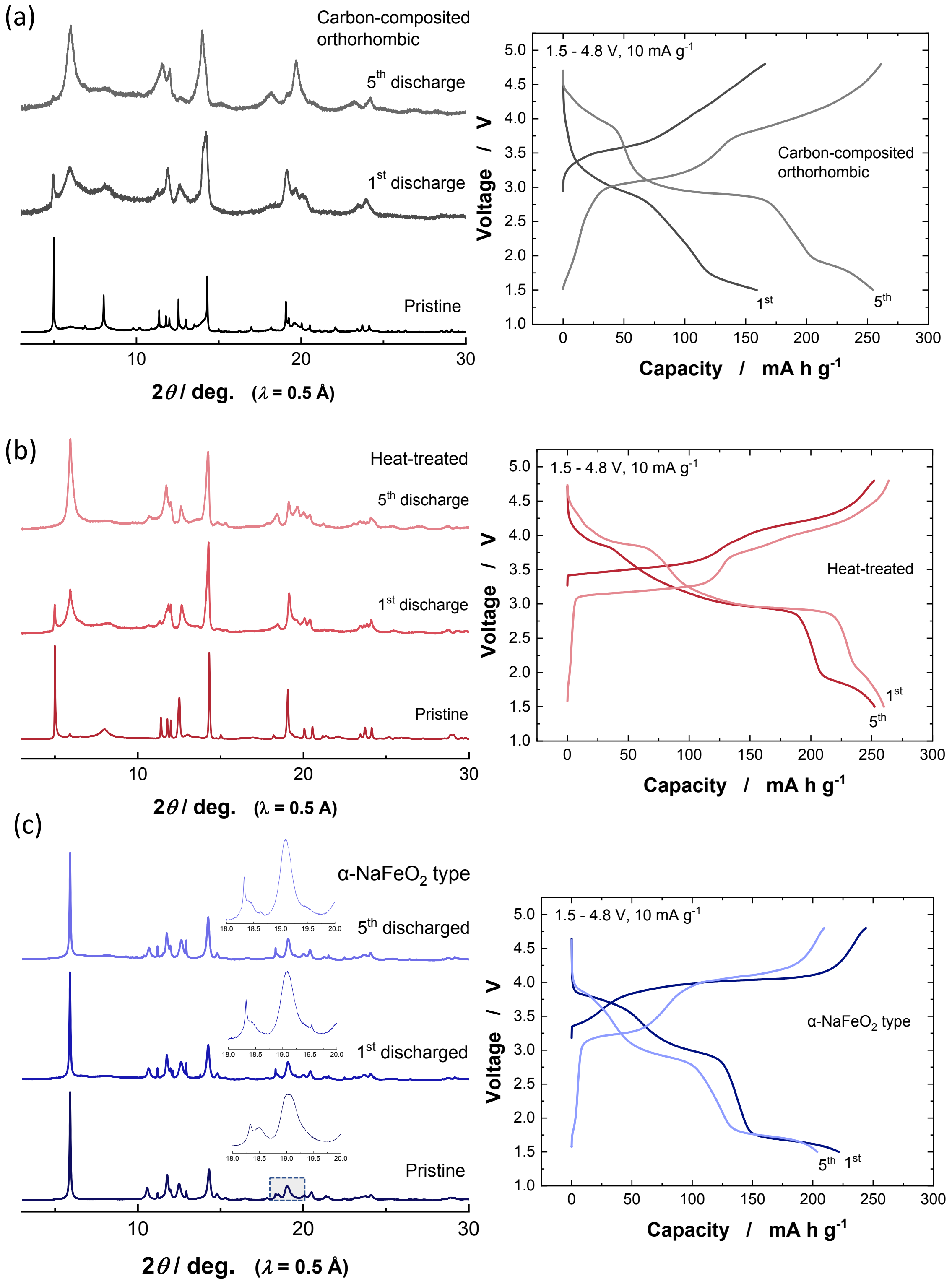

Figure S11. Structural evolution of different $LiMnO_2$ polymorphs. Corresponding charge/discharge curves are also shown.

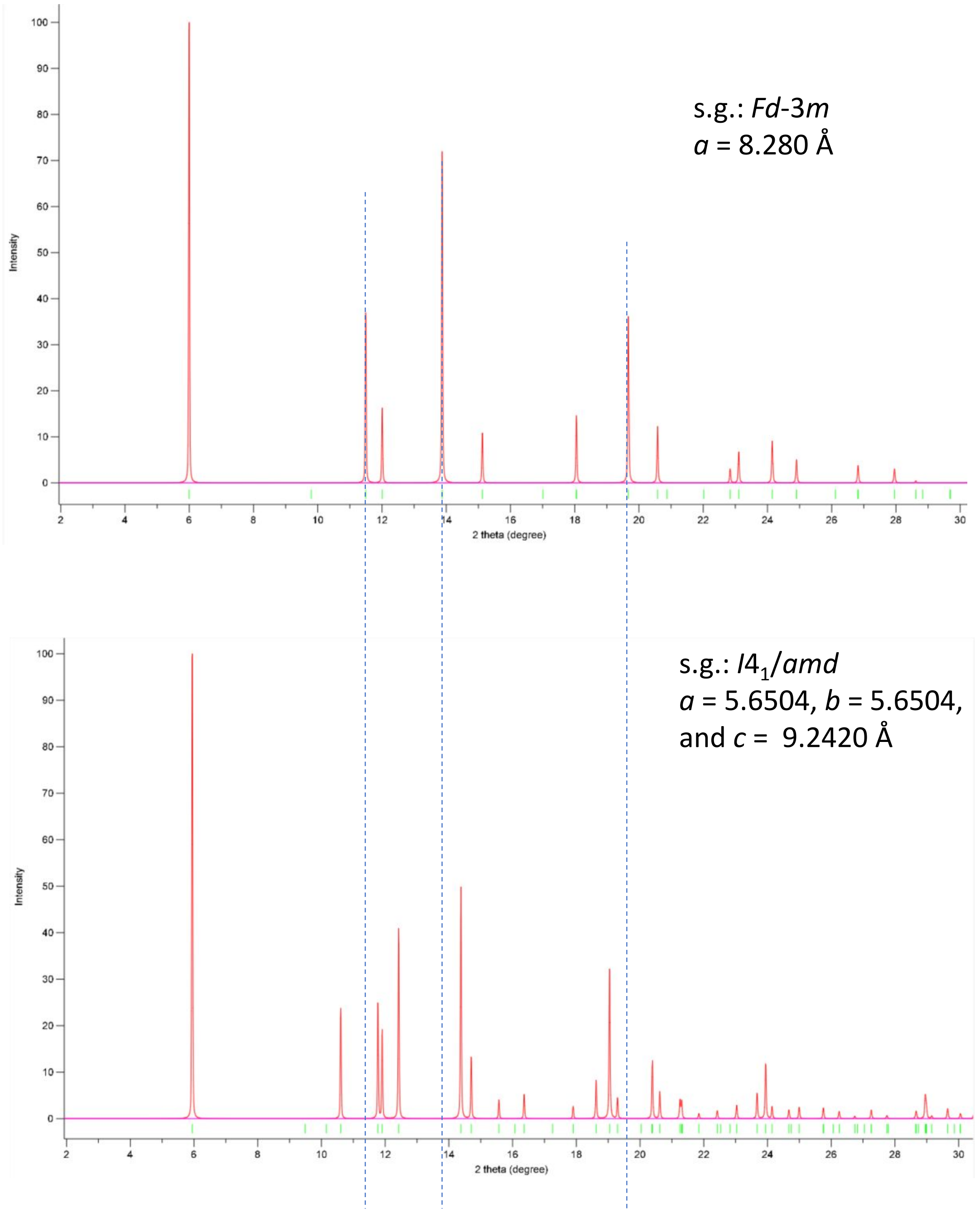

Figure S12. Simulated XRD patterns of cubic and tetragonal $Li_2Mn_2O_4$ ($Li_{1+x}Mn_2O_4$).

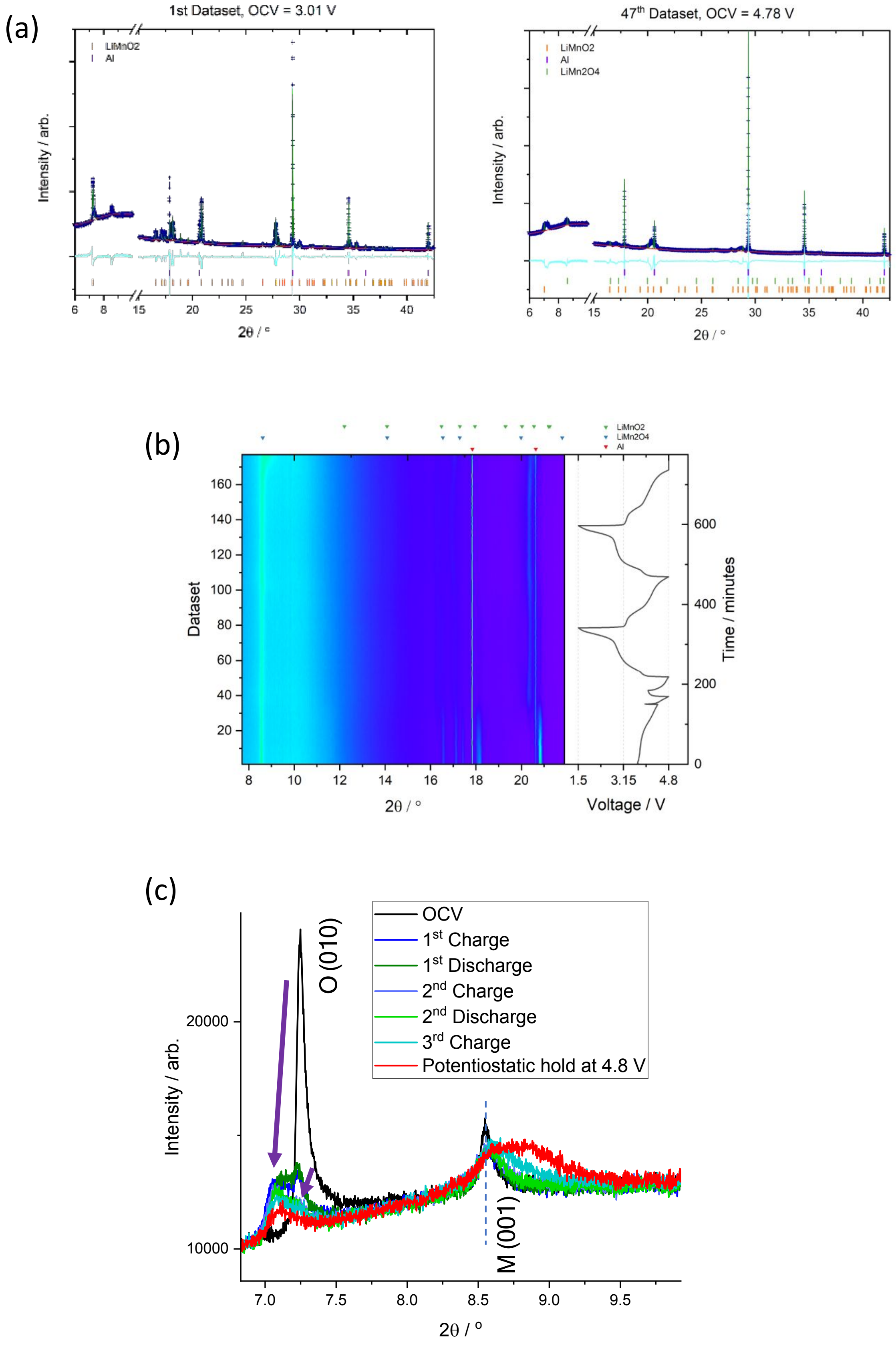
(a)
1st Dataset, OCV = 3.01 V
LiMnO2
Al
Intensity / arb.
2θ / °
47th Dataset, OCV = 4.78 V
LiMnO2
Al
LiMn2O4
Intensity / arb.
2θ / °
(b)
LiMnO2
LiMn2O4
Al
Dataset
2θ / °
Voltage / V
Time / minutes
(c)
OCV
1st Charge
1st Discharge
2nd Charge
2nd Discharge
3rd Charge
Potentiostatic hold at 4.8 V
O (010)
M (001)
Intensity / arb.
2θ / °

(d)

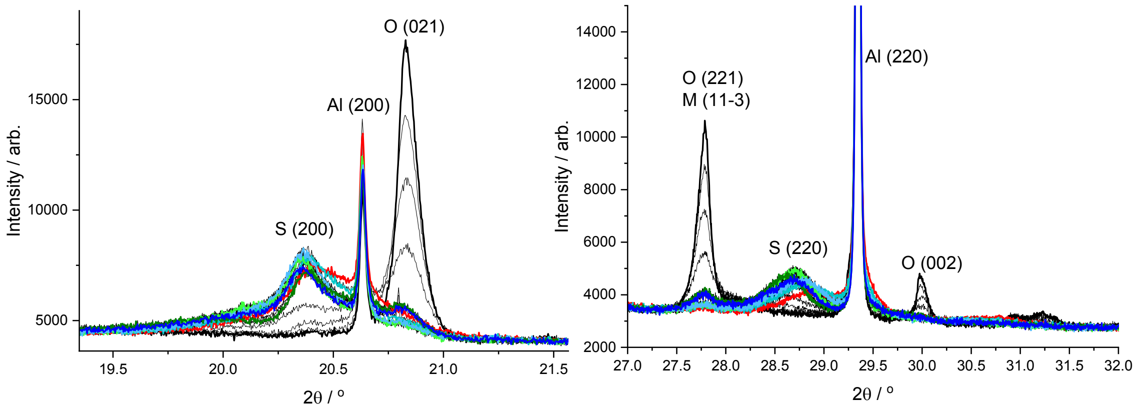

(e)

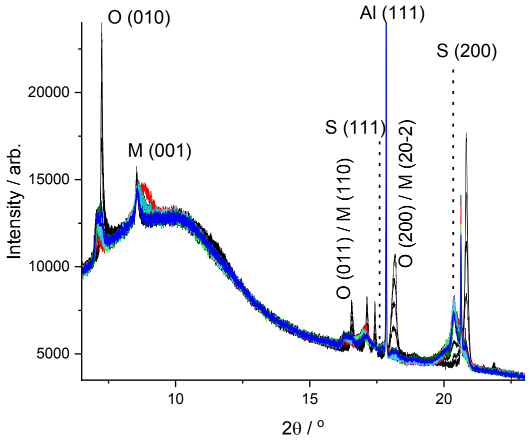

(f)

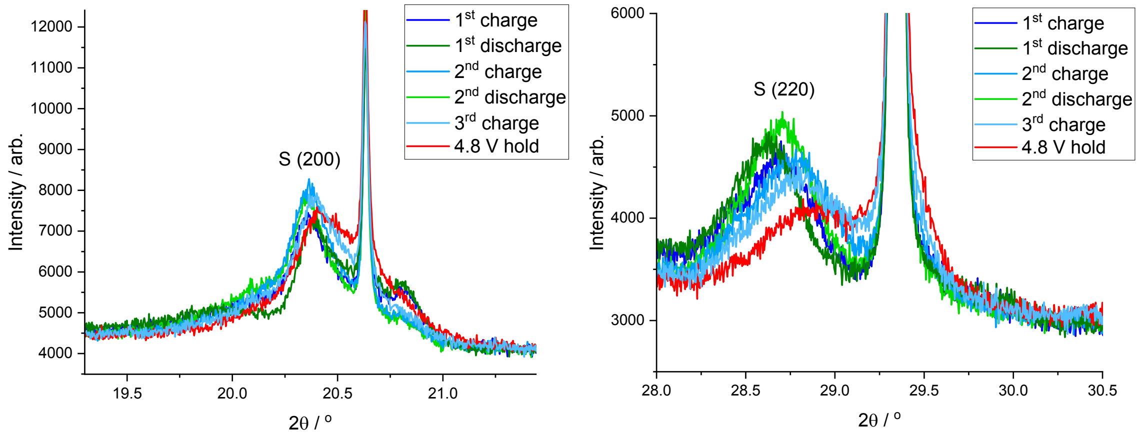

Figure S13. (a) Selected Rietveld-refined fits of structural models to the *operando* XRD data for heat-treated $LiMnO_2$, and the enlarged contour plot of *operando* XRD patterns (**Figure 3b**) is shown in (b). (c) Selected XRD patterns from the *operando* XRD experiment. On first charge,

the reduction in intensity of the orthorhombic (010) reflection is indicated by the longer purple arrow. The next reduction in intensity is observed on 2nd charge indicated by the smaller purple arrow. (d) Every tenth *operando* XRD pattern overlayed (in black and color scheme for charge/discharge/hold as in (c). (left) The starting electrode shows only the presence of the orthorhombic (021) reflection and upon 1st charge (dark blue) reduces in intensity and the spinel (S) (200) appears. The spinel-like reflection is maintained throughout subsequent cycling. (right) The starting electrode shows only the presence of the orthorhombic (221) and (002) reflections and upon 1st charge (dark blue) reduces in intensity and the spinel (S) (220) appears. The spinel-like reflection is maintained throughout subsequent cycling. (e) Every tenth pattern in the *operand*o XRD experiment overlayed. Every tenth operando XRD pattern overlayed (in black and color scheme for charge/discharge/hold as in (c). (f) The evolution of the 200 and 220 reflections of the spinel-like phase from the 1st charge to the 4.8 V hold step. The color scheme is maintained from (c).

(a)

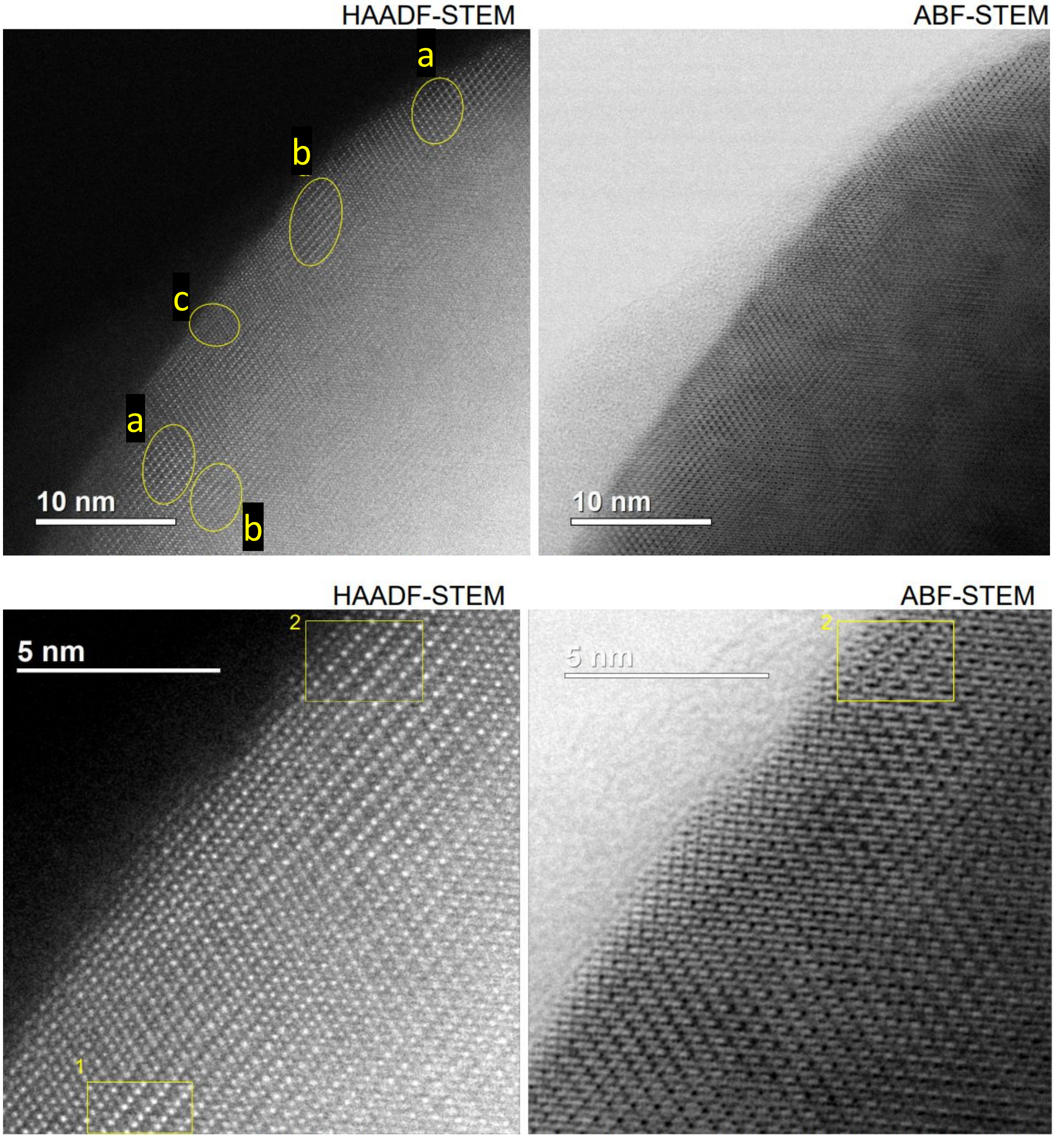
HAADF-STEM
ABF-STEM
a
b
c
a
b
10 nm
10 nm
HAADF-STEM
ABF-STEM
5 nm
5 nm
1
2
2

(b) HAADF-STEM BF-STEM

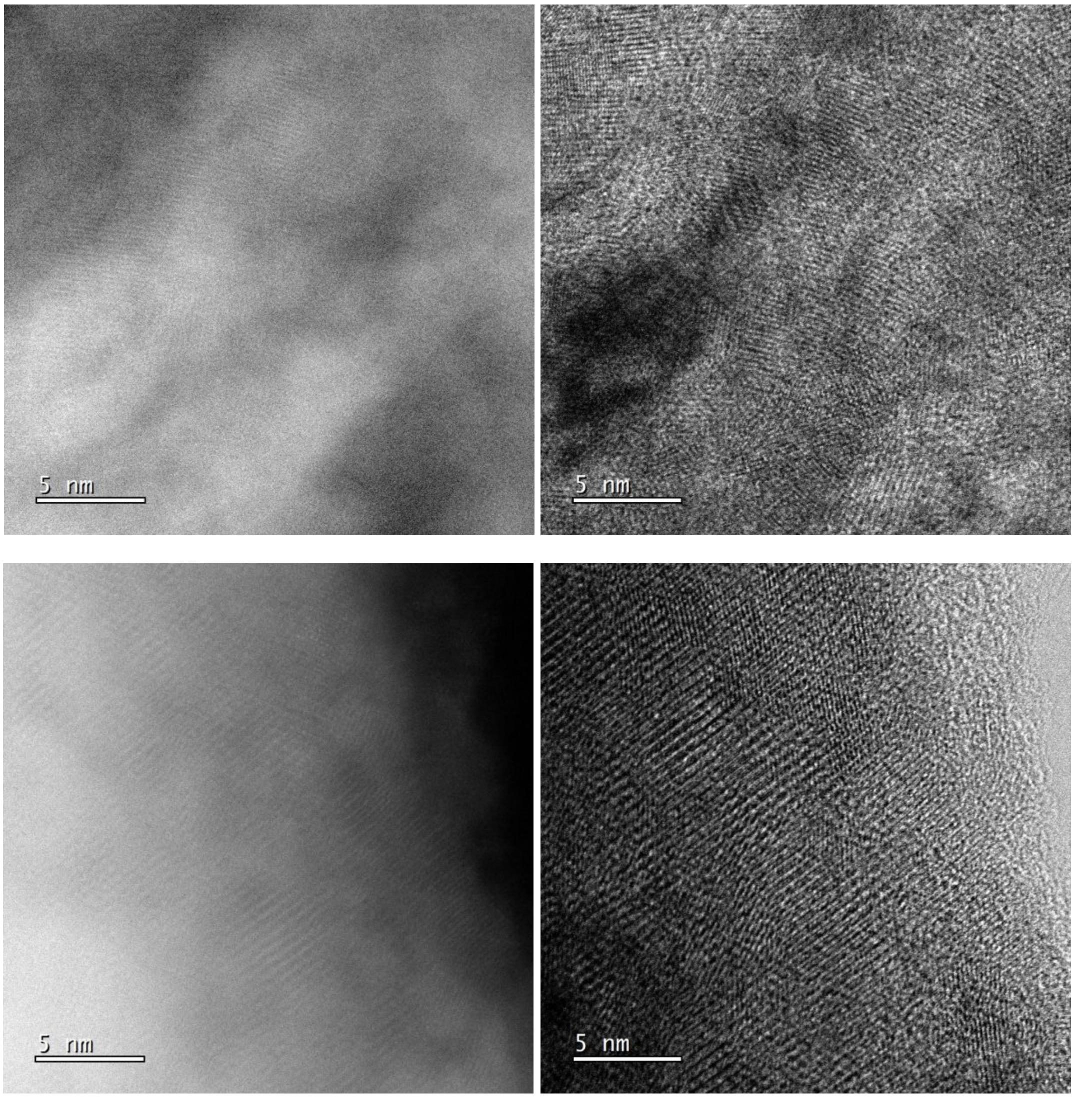

(c) HAADF-STEM BF-STEM

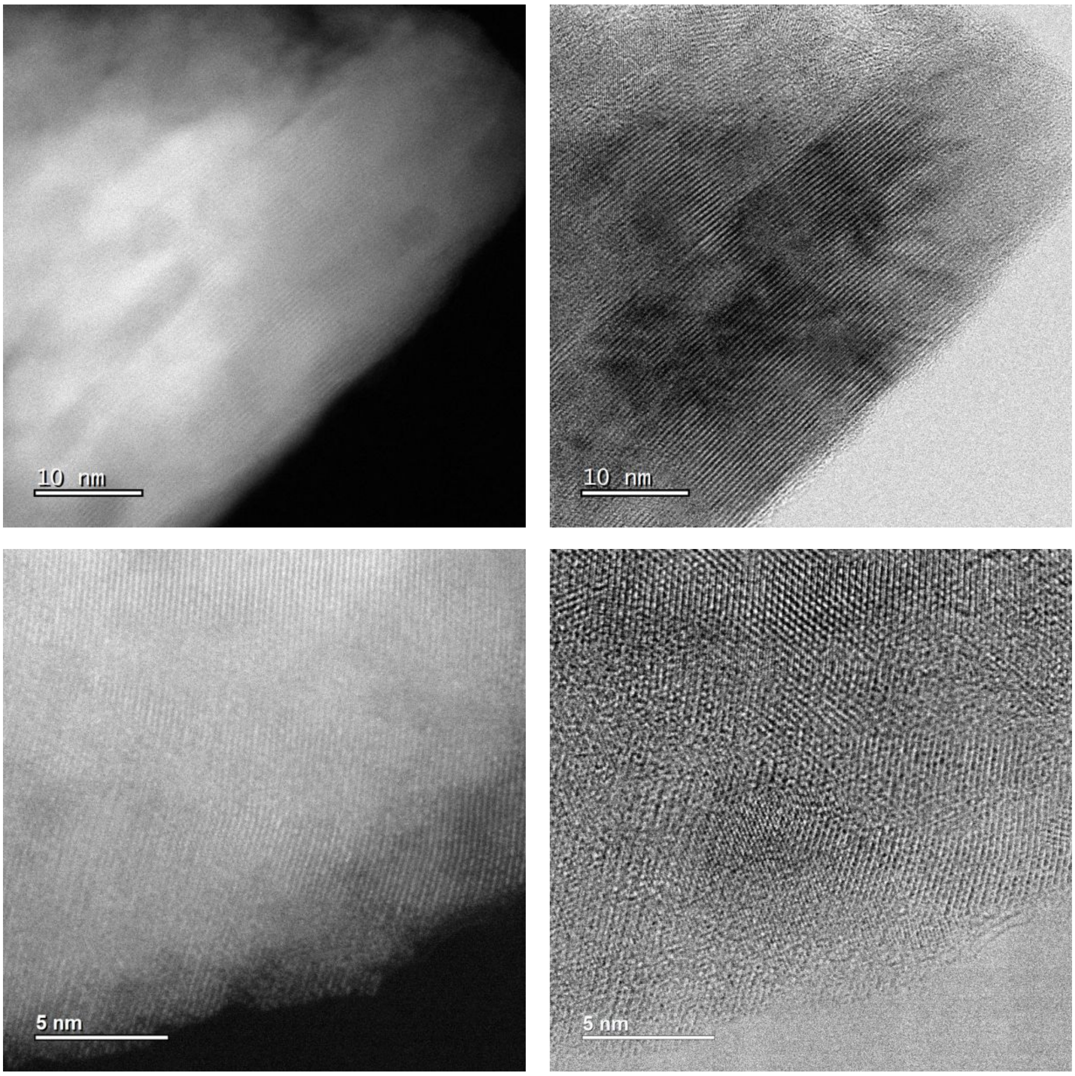

Figure S14. (a) STEM images of heat-treated $LiMnO_2$ after cycle test. The data of cycled samples for (b) orthorhombic and (c) monoclinic layered $LiMnO_2$ are also compared. Smaller nanosized domains are formed for the sample derived from the orthorhombic sample. In contrast, higher crystallinity regions with a layered-like structure are found for the sample derived from the monoclinic layered sample even though nanosized domains are similarly formed in different regions.

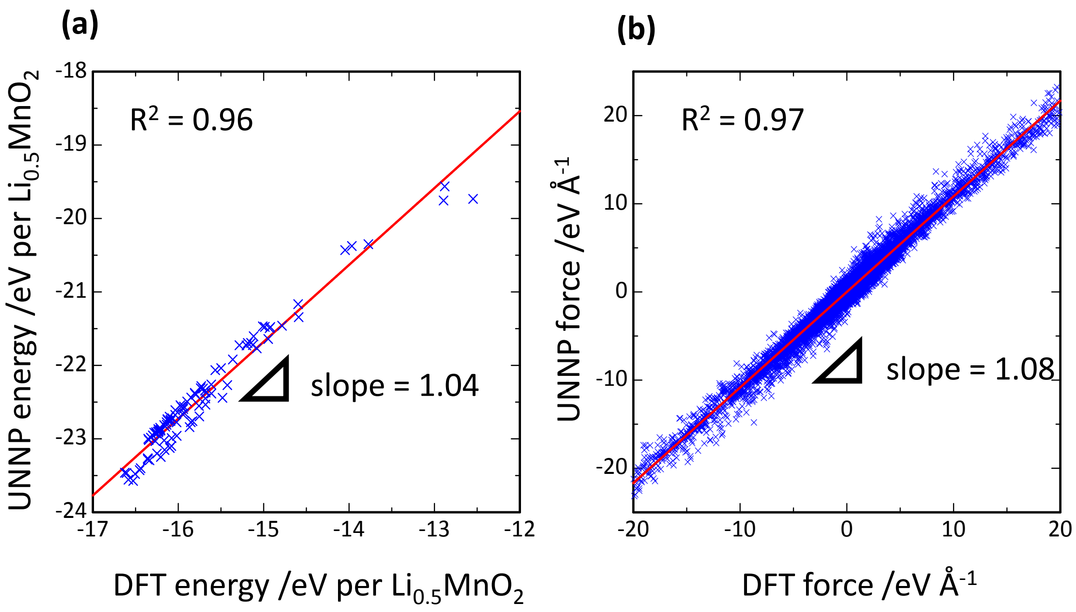

Figure S15. Diagnostic plots of (a) energies and (b) forces obtained by DFT and UNNP calculations using randomly distorted $Li_{0.5}MnO_2$ with layered and orthorhombic ordering. Red lines correspond to least square regression lines.

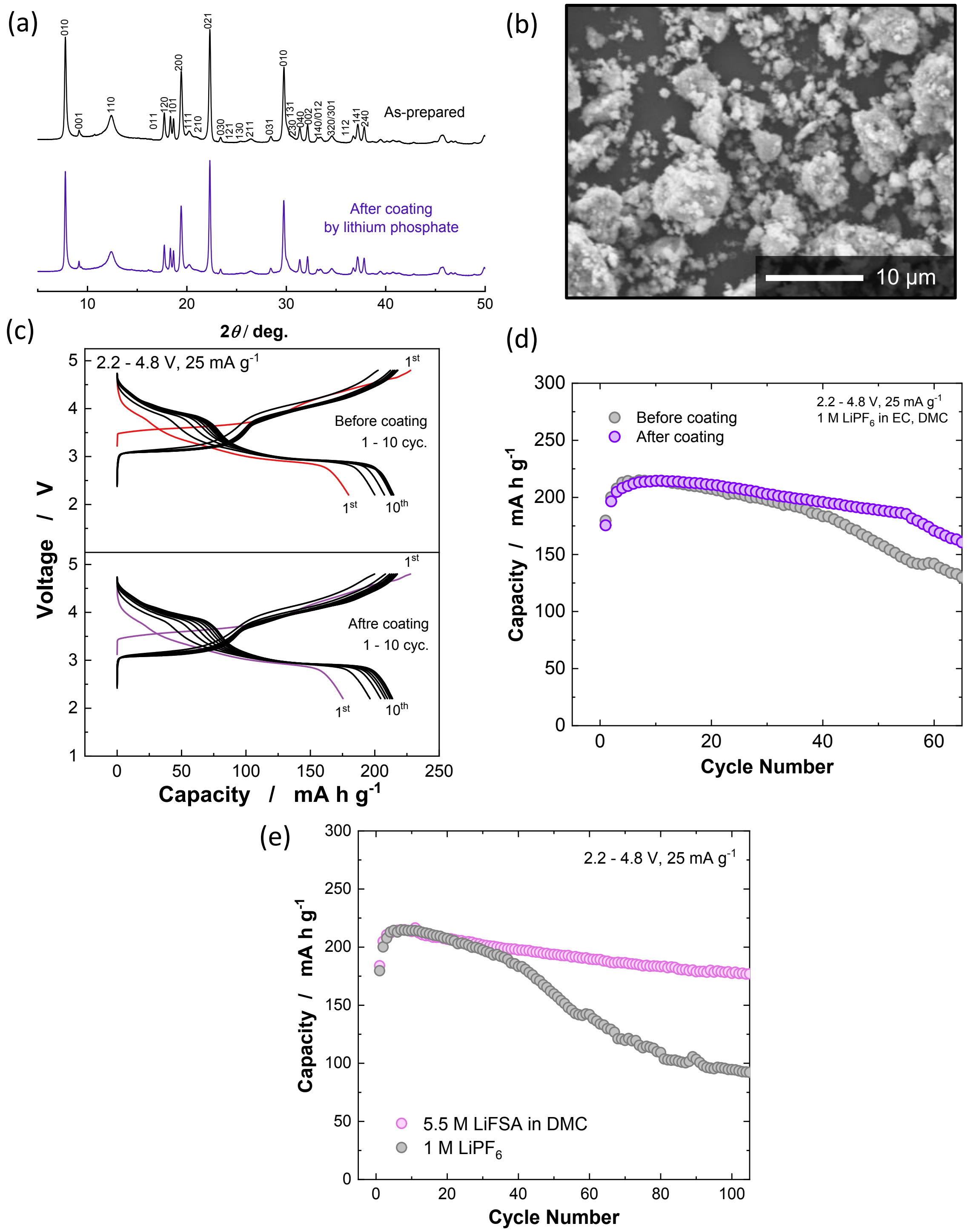

Figure S16. Characterization and electrochemistry of nanostructured $LiMnO_2$; (a) XRD patterns of samples with or without lithium phosphate coating, (b) a SEM image of as-preapred sample,

(c) charge/discharge curves and (d) capacity retention of the samples with or without lithium phosphate coating, (e) capacity retention of coated sample in 5.5 M LiFSA/DMC.

Supporting References

(1) Croguennec, L.; Deniard, P.; Brec, R.; Lecerf, A. Nature of the stacking faults in orthorhombic LiMnO2, *J. Mater. Chem.* **1997**, *7*, 511-516.

(2) Treacy, M. M. J.; Newsam, J. M.; Deem, M. W. A GENERAL RECURSION METHOD FOR CALCULATING DIFFRACTED INTENSITIES FROM CRYSTALS CONTAINING PLANAR FAULTS, *Proceedings of the Royal Society of London Series a-Mathematical Physical and Engineering Sciences* **1991**, *433*, 499-520.
